# Lessons learned from 20 years of preclinical testing in pediatric cancers

**DOI:** 10.1016/j.pharmthera.2024.108742

**Published:** 2024-11-05

**Authors:** Malcolm A. Smith, Peter J. Houghton, Richard B. Lock, John M. Maris, Richard Gorlick, Raushan T. Kurmasheva, Xiao-Nan Li, Beverly A. Teicher, Jeffrey H. Chuang, Filemon S. Dela Cruz, Michael A. Dyer, Andrew L. Kung, Michael W. Lloyd, Yael P. Mossé, Timothy M. Stearns, Elizabeth A. Stewart, Carol J. Bult, Stephen W. Erickson

**Affiliations:** a National Cancer Institute, Bethesda, MD, United States of America; b The University of Texas Health at San Antonio, TX, United States of America; c Children’s Cancer Institute, Lowy Cancer Research Centre, School of Clinical Medicine, UNSW Medicine & Health, UNSW Centre for Childhood Cancer Research, UNSW Sydney, Sydney, NSW, Australia; d The Children’s Hospital of Philadelphia and the Perelman School of Medicine at the University of Pennsylvania, Philadelphia, PA, United States of America; e The University of Texas MD Anderson Cancer Center, Houston, TX, United States of America; f Lurie Children’s Hospital, Northwestern University Feiberg School of Medicine, Chicago, IL, United States of America; g The Jackson Laboratory for Genomic Medicine, Farmington, CT, United States of America; h Memorial Sloan Kettering Cancer Center, New York City, NY, United States of America; i St. Jude Children’s Research Hospital, Memphis, TN, United States of America; j The Jackson Laboratory for Mammalian Genetics, Bar Harbor, ME, United States of America; k RTI International, Research Triangle Park, NC, United States of America

**Keywords:** Pediatric cancer, Preclinical testing, Targeted therapy, RACE act

## Abstract

Programs for preclinical testing of targeted cancer agents in murine models of childhood cancers have been supported by the National Cancer Institute (NCI) since 2004. These programs were established to work collaboratively with industry partners to address the paucity of targeted agents for pediatric cancers compared with the large number of agents developed and approved for malignancies primarily affecting adults. The distinctive biology of pediatric cancers and the relatively small numbers of pediatric cancer patients are major challenges for pediatric oncology drug development. These factors are exacerbated by the division of cancers into multiple subtypes that are further sub-classified by their genomic properties. The imbalance between the large number of candidate agents and small patient populations requires careful prioritization of agents developed for adult cancers for clinical evaluation in children with cancer. The NCI-supported preclinical pediatric programs have published positive and negative results of efficacy testing for over 100 agents to aid the pediatric research community in identifying the most promising candidates to move forward for clinical testing in pediatric oncology. Here, we review and summarize lessons learned from two decades of experience with the design and execution of preclinical trials of antineoplastic agents in murine models of childhood cancers.

## 1. Introduction

**Fig. 1.**
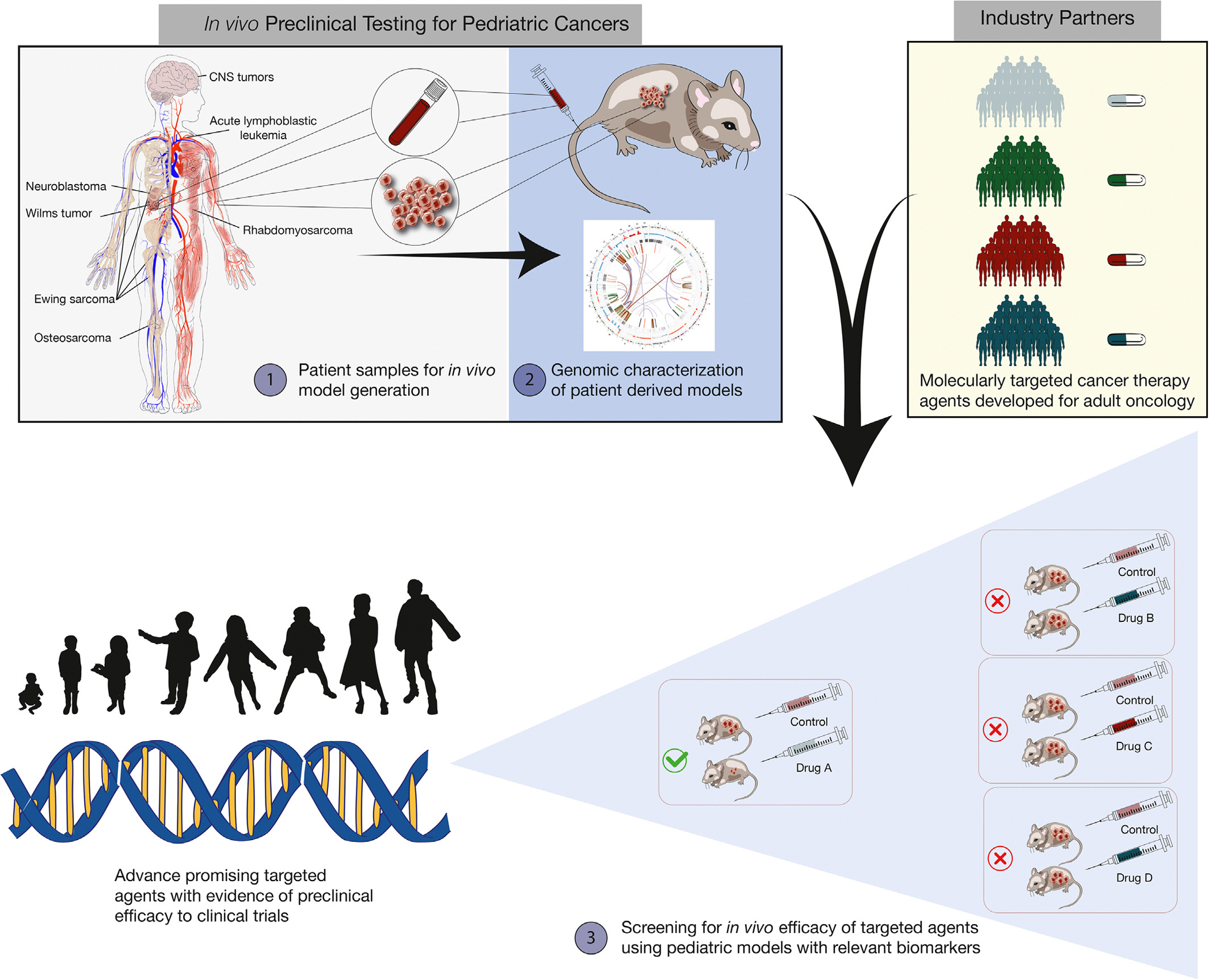
Patient-derived models established from patient tissue samples for diverse pediatric cancers are genomically characterized and selected for testing in collaboration with industry partners who have developed targeted cancer therapy agents for adult oncology. The *in vivo* study results identify the targeted therapies with promise for clinical application in pediatric oncology.

The Pediatric Preclinical Testing Program and its successor, the Pediatric Preclinical Testing Consortium (together termed the PPTP/C), were supported by NCI from 2004 through 2021. The current Pediatric Preclinical *In Vivo* Testing Consortium (PIVOT) is a continuation and expansion of these programs. The challenge that these pediatric preclinical testing programs were designed to address is the large number of agents entering clinical evaluation for adults with cancer compared to the thankfully relatively small number of children with specific cancer types against which these agents might be tested (Fig. 1). The challenges of small patient numbers are exacerbated by the genomic, epigenomic, and proteomic revolutions in cancer research, which led to the division of specific pediatric cancers into multiple biologically defined subtypes. This imbalance between a multitude of candidate agents and small patient populations creates the need to prioritize among the universe of agents in development for adult cancers those most warranting clinical evaluation in children with cancer.

The PPTP/C took a systematic and unbiased approach to evaluating candidate agents by testing for their activity across a range of pediatric cancer types. The PPTP also included an *in vitro* testing component, but this was not continued in the PPTC in favor of a strict focus on *in vivo* testing. Results from studies performed by the PPTP/C, both positive and negative, have been published so that the pediatric research community can access the results to assist in decision-making about agents to move forward for clinical testing. Here we provide a comprehensive review and assessment of the testing results and highlight the lessons that can be learned from the PPTP/C testing experience. In the following text, we summarize our experience with *in vivo* testing of more than 100 agents and draw lessons that can assist in designing more informative and efficient research plans for future testing efforts.

## 2. Proteoepigenomic characterization of preclinical models is critical for designing and interpreting preclinical efficacy data

At the initiation of the PPTP/C testing program in 2004, the first draft of the human genome had just been completed (International Human Genome Sequencing, 2004). Next generation sequencing (NGS) was subsequently developed with James Watson’s genome being the first published using NGS technology (Wadman, 2008). The Cancer Genome Atlas (TCGA) was launched at about the same time (Grobner et al., 2018), and pediatric cancer NGS projects focused on diagnostic tumor samples followed shortly thereafter (Ma et al., 2018). Thus, the first decade or more of PPTP/C testing was primarily performed with little knowledge of the key molecular drivers of the models being studied. Indeed, one of the Program’s initial goals was to uncover recurrent oncogenic drivers of childhood cancers revealed by high response rates of small molecular inhibitors of key pathways mutated in adult malignancies. As discussed below, most agents tested during this time showed low response rates, with occasional exceptional responses observed. This was explained in retrospect, at least in part, by the overall low mutation rates and corresponding small number of targetable genomic alterations observed in childhood cancers, a finding that became apparent as pediatric cancer genomic studies began to be published in the early 2010s. What these studies demonstrated, however, is that important therapeutically tractable molecular subsets do exist in some childhood cancers, and robust responses were observed in the right therapeutic context. It thus became essential to genomically characterize the patient-derived xenograft (PDX) models used in the PPTP/C to allow for rational preclinical trial design matched to the mechanism of action of the agent under study.

A comprehensive genomics characterization effort was launched in early 2016, supported by Alex’s Lemonade Stand Foundation. This resulted in whole exome sequencing, RNA sequencing and high-density single nucleotide polymorphism array data being generated from 261 models from 37 unique pediatric cancer histologies studied in the PPTP/C (Rokita et al., 2019). Important lessons from this effort included: 1) the models used in the Program do faithfully recapitulate the genomic alterations seen in the childhood cancers under study; 2) overall mutation burden was generally higher across histologies, presumably due to the fact that many relapse or post-mortem samples were used to establish the models; and 3) gene expression signatures also faithfully recapitulated those seen in primary tumors. All 261 models have been entered into the NCI-funded Patient-Derived Cancer Models (PDCM) database (https://www.cancermodels.org/) with the goal of making these models and associated information available to academically qualified petitioners and/or the pharmaceutical industry (Perova et al., 2023). Of note, similar genomic characterization efforts were undertaken by multiple other groups at this time and reached similar, if not identical, conclusions (Brabetz et al., 2018; Jones et al., 2019; Stewart et al., 2017).

Despite the success of these programs, several gaps remain. First, while the genomic architecture of the models does recapitulate what is seen in patient samples, they do not maintain subclonal heterogeneity, which is likely a major mediator of therapy resistance. High-risk neuroblastomas, for example, show an admixture of adrenergic and mesenchymal cells that are epigenetically determined (Boeva et al., 2017; van Groningen et al., 2017). However, the neuroblastoma PDX models assume a completely adrenergic transcriptional profile (Maris Lab, unpublished observations), likely due to the more proliferative adrenergic cells outcompeting the more quiescent mesenchymal cells. Further, genomic profiling of pediatric patient solid tumors and their matching PDX models reveals an interplay between intratumor heterogeneity and immune constraints on tumor evolution (He et al., 2023). In addition, while quantification of mRNA abundance by RNA sequencing often predicts protein abundance, it has been estimated that 50 % of the time there is discordance (Ghazalpour et al., 2011). This is critically important as more immunotherapeutic strategies targeting surface proteins move into pediatric preclinical testing. Finally, epigenetic characterization of the models is incomplete, which is critically important as more diseases and therapy decisions are being based on methylation profiles or other epigenetic states. Thus, as more models are continuously generated from children with cancer across the spectrum of their therapeutic journeys, the pediatric cancer community must prepare for the continuous proteogenomic and epigenetic characterization of models used to make important decisions about which drugs in development are tested in children with cancer in a robust biomarker-directed fashion.

## 3. Summary of two decades of preclinical testing in pediatric models

A stated goal for establishing the PPTP/C was to develop an approach to preclinical testing that would help guide pediatric drug development by informing prioritization decisions for selecting among the hundreds of agents in development for adults with cancer those agents that warranted evaluation in children (Houghton et al., 2002). As the ultimate objective for bringing a new agent into evaluation in the pediatric population is curing more children, and as the pathway to cure involves eliminating cancer cells, the PPTP/C has focused on identifying agents able to induce tumor regressions, and ideally maintained complete responses. The history of combination therapy in pediatric oncology has been built around designing combinations that use agents that each have robust tumor-regressing activity (Plana, Palmer, & Sorger, 2022), and this practice is supported by analyses showing that additivity predicts the efficacy of most approved combination therapies used to treat advanced cancers (Hwangbo, Patterson, Dai, Plana, & Palmer, 2023; Palmer, Izar, Hwangbo, & Sorger, 2022). The high levels of tumor-regressing activity for standard-of-care agents against PPTP/C models supports the concept that agents with *in vivo* activity in pediatric preclinical models may be effective in the clinic (Houghton et al., 2007). The converse, that agents that are found to be inactive in *in vivo* preclinical testing can be deprioritized because of a low likelihood that the agents will have meaningful activity, is an important corollary that the PPTP/C evaluated. Because the PPTP/C was initially envisioned as an experiment with a goal of determining the predictive value of preclinical models, it is not surprising that some agents with little or no tumor-regressing *in vivo* activity in PPTP/C testing proceeded to clinical testing. More importantly, going forward the PPTP/C experience supports waiving clinical evaluations in children of agents that have tumor-cell intrinsic mechanisms of action that show limited tumor regressing activity in an appropriate set of pediatric preclinical models.

The primary method used by the PPTP/C to assess anticancer activity used objective response categories modeled after those used in the clinic. Response categories indicating tumor regression for solid tumor models (or remission for leukemia models) were maintained complete response (MCR), complete response (CR), and partial response (PR). In following clinical trial conventions, models achieving MCR, CR, or PR are grouped together in reporting the objective response rate (ORR). Two response categories were used to describe tumor progression: progressive disease 1 (PD1) and progressive disease 2 (PD2), with the former indicating progressive disease with less than a 2-fold prolongation of time to event and the latter indicating progressive disease with greater than 2-fold prolongation in time to event relative to vehicle controls. As described below, the PD2 category was primarily useful in describing the activity of VEGF-pathway targeting agents. Because a primary focus of the PPTP/C was to identify agents able to induce robust regressions for solid tumors and remissions for leukemias, the discussion of agent activity that follows focuses on the ORR of tested agents. Defining activity based on the ability of agents to induce tumor regressions is different from the benchmark used in some preclinical testing reports in which a significant slowing of tumor growth is considered to indicate potentially clinically meaningful activity. This distinction is important in explaining the relatively small numbers of agents identified as “active” by the PPTP/C. Detailed statistical analysis methods and response metrics for each agent tested by the PPTP/C are available from the Zenodo research repository (DOI: https://doi.org/10.5281/zenodo.13871579).

Agents were typically tested against 40 to 50 preclinical models, including tumor panels for acute lymphoblastic leukemia (ALL), osteosarcoma, neuroblastoma, rhabdomyosarcoma, Ewing sarcoma, Wilms tumor, rhabdoid tumors, and selected central nervous system (CNS) tumors. Based on their mechanism of action, some agents were tested against a limited set of models representing one or more selected tumor types.

Contrary to the perception among some cancer researchers that most agents tested preclinically show activity against xenograft-bearing mouse models, only 21 % of testing experiments using PPTP/C models resulted in an objective response across all agents tested. The ORR differed between solid tumor models and acute lymphoblastic leukemia models, being only 15 % for the solid tumor models and 37 % for the ALL models, again across all standard-of-care and new agents tested (Table 1).

### 3.1. HDAC, HSP90, and proteasome inhibitors exemplify classes of targeted agents with limited tumor-regressing activity across a broad range of pediatric cancers

Three classes of agents that showed minimal *in vivo* activity in PPTP/C testing were inhibitors of HSP90, HDAC, and the proteasome. Agents in the HDAC and proteasome inhibitor classes have achieved regulatory approval for hematologic malignancies (multiple myeloma and selected lymphomas) but not for solid tumors (Kambhampati & Wiita, 2020). For HSP90 inhibitors, despite 17 agents entering clinical evaluation through 2020, none have achieved regulatory approval (Cappellacci, Perinelli, Maggi, Grifantini, & Petrelli, 2020; Koren & Blagg, 2020). These agent classes share two common characteristics: targets that are ubiquitously expressed and that lack predictive biomarkers to guide selection of target tumors. Focusing on PPTP/C solid tumor testing results, the ORRs for three HSP90 inhibitors (Kang et al., 2012; Lock et al., 2013; Smith et al., 2008), three HDAC inhibitors (Carol et al., 2014; Keshelava et al., 2009; Kurmasheva et al., 2019), and a single proteasome inhibitor (Houghton et al., 2008) were 3 %, 1 %, and 6 %, respectively (Table 2).

Turning to clinical results, for the HSP90 inhibitor tanespimycin, two pediatric clinical trials enrolled 32 patients, with no patients achieving an objective response (Bagatell et al., 2007; Weigel et al., 2007). A pediatric phase 1 trial of bortezomib in children with solid tumors observed no responses in 11 assessable patients. A phase 1 trial of vorinostat evaluated as a single agent and in combination with 13-cis retinoic acid (13cRA) in children with refractory solid tumors also observed no objective responses among 29 patients treated with single agent vorinostat and only one objective response among 24 evaluable patients treated with vorinostat and 13cRA (a patient with evaluable disease detected by ^123^I-metaiodobenzylguanidine (^123^I-MIBG) at study entry and after course 9 had no abnormal radiotracer uptake) (Fouladi et al., 2010). A clinical trial for the HDAC inhibitor vorinostat observed no responses in 21 children who started therapy (Bukowinski et al., 2021). However, vorinostat has been shown to increase the expression of the norepinephrine transporter, which is the target and path of entry for radiolabeled MIBG into neuroblastoma cells (More et al., 2011), and a randomized phase 2 trial of ^131^I-MIBG targeted radiotherapy with or without vorinostat showed objective response rates of 14 % and 32 %, respectively, in a “pick-the-winner” trial design (DuBois et al., 2021). The PPTP/C testing of vorinostat was not designed to detect this type of potentially synergistic clinical activity, and further clinical evaluation is needed to confirm the benefit of vorinostat added to ^131^I-MIBG.

For each of the agents described above that proceeded to clinical trials in children, there was some preclinical rationale to support their evaluation. Often the preclinical rationale focused on *in vitro* activity, and each of the agents in these three classes showed *in vitro* activity against the pediatric cell lines utilized by the PPTP/C. The potency of the agents ranged from low nanomolar IC_50_ values for agents like bortezomib (Houghton et al., 2005), ganetespib (Lock et al., 2013), and quisinostat (Carol et al., 2014) to micromolar level IC_50_ values for vorinostat (Keshelava et al., 2009). The disconnect between *in vitro* activity and clinical activity is exemplified by the observation that multiple myeloma cell lines were not the most sensitive to proteasome inhibition in a large cancer cell line panel, with melanoma, renal carcinoma and glioblastoma cell lines showing greater sensitivity (Kambhampati & Wiita, 2020). One factor contributing to the failure of *in vitro* testing results to predict clinical activity is the difficulty in benchmarking (or failure to benchmark) drug exposures effective *in vitro* against cancer cell lines with the drug exposures that can be tolerated by the wide range of normal tissues for which toxic effects may limit drug dosing (Smith & Houghton, 2013). Another factor contributing to the limited predictive value of cell lines are the changes in cell biology required for cells to adapt to 2-dimensional culture, such that key characteristics of the cancer cells in patients may be absent in cultured cell lines (Kambhampati & Wiita, 2020). The utility of organoids and other 3-dimensional *ex vivo* models in predicting clinical activity of cancer therapeutics is an area of active ongoing investigation (Driehuis, Kretzschmar, & Clevers, 2020; Drost & Clevers, 2018; Wood & Ewald, 2021).

For agents lacking biomarkers that are known to predict clinical activity, the PPTP/C experience with HDAC, proteasome, and HSP90 inhibitors (as well as other agents showing limited *in vivo* activity in pediatric preclinical models) supports the approach of considering *in vitro* activity for an agent as supportive data but with *in vivo* activity as highly desirable (if not mandatory) for proceeding to clinical evaluations of agents in children.

### 3.2. VEGF pathway inhibitors slow tumor growth but rarely cause objective responses in preclinical models, consistent with their clinical effect

The PPTP/C studied six different VEGF-pathway targeted agents: cediranib (Morton et al., 2012), sunitinib (Maris et al., 2008), sorafenib (Keir et al., 2010), pazopanib (Keir et al., 2012), regorafenib (Harrison et al., 2020), and cabozantinib (Smith et al., 2013). These agents range from those with somewhat greater relative selectivity for VEGFR2 signaling (*e.g.*, cediranib) to agents classified as multi-targeted kinase inhibitors for which VEGFR2 is one of multiple kinases that are potently inhibited (*e.g.*, sunitinib, sorafenib, regorafenib, and cabozantinib). For four of the agents tested (cediranib, sunitinib, sorafenib, and cabozantinib), *in vitro* testing against a 23-cell line panel was conducted. The *in vitro* findings were consistent across the agents tested, with a single cell line with an activating *KIT* mutation (the AML cell line Kasumi-1) showing *in vitro* sensitivity at 1–2 log lower concentrations than the remaining cell lines (Keir et al., 2010; Maris et al., 2008; Morton et al., 2012; Smith et al., 2013). This observation is consistent with these kinase inhibitors having little or no intrinsic anticancer activity against the cell lines and with the *in vivo* effects of these agents reflecting their impact on the tumor microenvironment rather than a cancer cell-directed effect.

The *in vivo* activity of the VEGF-pathway targeted agents against solid tumor models was notable for the consistent pattern of slowing of tumor growth with tumor regressions being uncommon. Only 4 % of the solid tumor models showed an objective response to the six VEGF-pathway targeted agents, while 41 % of the models showed the 2-fold or greater slowing of tumor growth required for the PD2 response category (Table 3). This result is markedly different from the response results obtained for all non-VEGF pathway targeted agents for which a lower percentage showed PD2 responses (only 12 % of models) and a higher percentage showed an objective response (16 % of models) (Table 3).

Three of the VEGF-pathway targeted agents (cediranib, sorafenib, and sunitinib) were also tested *in vivo* against pediatric ALL models. In contrast to the results with solid tumor models, only 13 % of the ALL models showed a PD2 response (Table 3). An objective response among the ALL models was observed in the ALL-2 model that harbors a mutation in the first amino acid of the FLT3 juxtamembrane domain (Y572S, 0.62 VAF) that is predicted to be activating (Dolai et al., 2016), and it showed a PR to sunitinib (Maris et al., 2008). FLT3 is one of the kinases targeted by sunitinib (O’Farrell et al., 2003). These results support the conclusion that the VEGF-pathway targeted agents have no activity for the pediatric ALL models in the absence of a relevant kinase mutation.

The PPTP/C results for VEGF pathway targeted inhibitors align with the clinical experience for this class of agents. Multiple agents in this class (including sunitinib, sorafenib, pazopanib, and cabozantinib) are Federal Drug Administration (FDA)-approved for the treatment of renal cell carcinoma. Clear cell renal cell carcinoma (ccRCC) is one of the few adult cancers for which the VEGF-pathway targeted inhibitors consistently show substantial objective response rates, and it is characterized by loss of *VHL* gene product function (Shulman, Shi, & Zhang, 2021). VHL is a component of the E3 ubiquitin ligase that binds to hypoxia-inducible factor (HIF) leading to HIF degradation, and loss of VHL function in ccRCC results in HIFα activation which drives VEGF expression (Gnarra et al., 1996). There is no known pediatric cancer correlate of ccRCC for which VHL loss of function is a key tumorigenic event. Several VEGF pathway inhibitors are also approved for hepatocellular carcinoma and regorafenib is approved for colorectal cancer. For these indications, the VEGF pathway inhibitors generally show low objective response rates, but significantly slow the rate of tumor progression in patients with advanced disease leading to prolongation of progression-free survival and/or overall survival (Bruix et al., 2017; Grothey et al., 2013; Llovet et al., 2008).

Multiple VEGF-pathway inhibitors have been tested in children (Akshintala et al., 2021; Chuk et al., 2018; Dubois et al., 2011; Fox et al., 2010; Gaspar et al., 2021; Geller et al., 2018; Geoerger et al., 2016; Glade Bender et al., 2013; Widemann et al., 2012). Objective responses have been uncommon in pediatric trials of VEGF-pathway targeted agents, and apparent slowing of tumor progression has been observed for some patients. Randomized phase 2 trials have been performed for lenvatinib and bevacizumab in pediatric patients with relapsed/refractory osteosarcoma and medulloblastoma, respectively (Gaspar et al., 2022; Levy et al., 2021). For each trial the anti-angiogenic agent was combined with standard chemotherapy. Slowing of time to progression was observed in both clinical trials but was statistically significant only in the bevacizumab study. The clinical experience in pediatric patients for VEGF-pathway targeted agents has mirrored the preclinical activity for these agents in the pediatric cancer models used by the PPTP/C.

### 3.3. Agents targeted to specific gene products (e.g., mutated kinases) rarely cause regressions in the absence of specific genomic alterations associated with sensitivity to the agent

A hallmark of cancer is the conferral of a selective growth and proliferative advantage, which can be brought about by the expression of mutated protein kinases and kinase gene fusions resulting in aberrant activation of intracellular signaling pathways (Fouad & Aanei, 2017; Hanahan, 2022; Hanahan & Weinberg, 2011). This is also true for pediatric malignancies, which can present with genomic lesions resulting in driver mutations in oncogenic protein kinases and activating fusions in signaling pathway genes (Grobner et al., 2018; Ma et al., 2018; Rokita et al., 2019). For example, germline mutations of the ALK receptor tyrosine kinase (*ALK*) gene are involved in the etiology of a subset of neuroblastomas (Mosse et al., 2008) and somatic *ALK* gain-of-function mutations, gene amplifications, and gene fusions are oncogenic drivers and therapeutic targets for neuroblastoma and anaplastic large cell lymphoma (Mosse et al., 2017). Activating mutations in *BRAF* are frequent in certain subtypes of pediatric brain tumors (Petralia et al., 2020). Moreover, activating mutations in *NOTCH1* occur in up to 75 % of T-ALL (Liu et al., 2017), the *BCR::ABL1* fusion is the oncogenic driver of Philadelphia chromosome-positive (Ph^+^) ALL (Bernt & Hunger, 2014), rearrangements of *KMT2A* involving multiple fusion partners are drivers of most cases of infant ALL as well as many cases of childhood AML (Meyer et al., 2023), and *FLT3* activating mutations are observed in leukemias (Grobner et al., 2018; Ma et al., 2018). Therefore, the PPTP/C tested numerous agents targeted to the protein products of specific genomic lesions that drive pediatric cancers against PDX models of pediatric cancers expressing those alterations. These agents included dasatinib (BMS-354825, SRC/ABL kinase inhibitor), selumetinib (AZD-6244/ARRY-142886, MEK1/2 (MAP2K1/2) inhibitor), sunitinib (SU11248, multi-targeted RTK inhibitor), SGI-1776 (PIM1/2/3 kinase inhibitor), AZD1480 and ruxolitinib/INCB18424 (JAK1/2 inhibitors), TAK-659 (dual SYK/FLT3 inhibitor), VTP-50469 (menin inhibitor) and talazoparib (BMN 673, PARP inhibitor). Those agents that demonstrated notable activity against the PPTP/PPTC pediatric cancer PDX models are discussed in greater detail below. Activity for these targeted agents is provided in Table 4.

#### 3.3.1. Dasatinib (BMS-354825)

The SRC/ABL kinase inhibitor dasatinib was tested against 43 solid tumor and ALL PDX models on a twice daily (once daily for the ALL models) x 5 schedule for an intended 4 weeks at a dose of 50 mg/kg (Kolb et al., 2008b). While dasatinib significantly delayed the progression of 11 PDXs relative to control vehicle-treated mice, the only PDX that sustained a prolonged regression was the ALL-4 PDX that harbored a *BCR::ABL1* fusion. Dasatinib is active in children and adults with Ph^+^ ALL harboring a *BCR::ABL1* fusion and is now commonly used in combination with standard chemotherapy to treat children with Ph^+^ ALL (Shen et al., 2020).

#### 3.3.2. Selumetinib (AZD-6244/ARRY-142886)

Selumetinib, a MEK1/2 inhibitor, was tested against 46 solid tumor and ALL PDXs on a twice daily x 5 and once daily x 2 schedule for an intended 6 weeks at a dose of 100 mg/kg (Kolb et al., 2010). The overall single agent response was limited, with only 11 PDXs exhibiting significant progression delay (including none of the ALL PDXs) relative to control vehicle-treated mice, albeit with slightly greater progression delay in the osteosarcoma and glioblastoma PDX models. One notable exception was a pilocytic astrocytoma PDX (BT-40) that harbored the well-characterized BRAF-activating mutation V600E. This model exhibited prolonged regression in response to multiple different schedules of selumetinib administration. Selumetinib has subsequently been shown to have clinical activity in children with low-grade glioma whose tumors have *BRAF* gene alterations (Fangusaro et al., 2019).

#### 3.3.3. Sunitinib (SU11248)

A total of 45 PDXs (37 solid tumor, 8 ALL) were evaluated against this multi-targeted kinase inhibitor, which was administered daily for an intended 28 days at a dose of 53.5 mg/kg (Maris et al., 2008). Significant progression delays relative to control vehicle-treated mice were observed in 22 PDXs (19 solid tumor, 3 ALL), although tumor regressions were only observed in a rhabdoid PDX (KT-16) and an ALL PDX (ALL-2). While the KT-16 PDX response remains unexplained, as mentioned above, ALL-2 harbors a mutation in the first amino acid of the FLT3 juxtamembrane domain (Y572S, 0.62 VAF) that is predicted to be activating and was associated with high FLT3 mRNA expression in this model (Dolai et al., 2016). The *in vivo* efficacy of sunitinib against ALL-2 contrasts with the lack of significant activity against ALL-3, a PDX that harbors a *KMT2 A* gene fusion and expresses high levels of *FLT3* mRNA, suggesting that high FLT3 expression alone is not sufficient for *in vivo* activity of sunitinib.

#### 3.3.4. VTP-50469

The menin inhibitor VTP-50469 was evaluated against a total of 16 PDX models (7 solid tumor, 9 ALL) administered twice daily for an intended 28 days at a dose of 120 mg/kg (Krivtsov et al., 2019; Kurmasheva et al., 2020). While the activity of VTP-50469 was limited against the solid tumor PDXs (significant progression delay observed in 4/6 models with no regressions) profound and prolonged regressions were observed in 6 ALL PDXs, including 6/8 harboring *KMT2A* rearrangements. The profound activity of VTP-50469 was predicted by previous work demonstrating the requirement for menin for leukemia induction by *KMT2A* gene fusions (Yokoyama et al., 2005; Yokoyama & Cleary, 2008). This on-target activity resulted in 9 mice remaining disease-free at around 1.5 years following the cessation of treatment, as well as another 5 mice that achieved remissions of close to one year, with no evidence of human leukemia in all 14 mice at euthanasia. The specificity of VTP-50469 against *KMT2A*-rearranged leukemia was further reinforced by its inability to significantly delay the progression of a *BCR::ABL1* fusion-positive ALL PDX (Krivtsov et al., 2019). Menin inhibitors have entered clinical evaluation and have shown remission-inducing activity in some patients with *KMT2A* fusion leukemias (Issa et al., 2023; McGeehan, 2020).

#### 3.3.5. Talazoparib (BMN 673)

The PARP inhibitor talazoparib was tested as a single agent against 44 pediatric cancer PDX models (36 solid tumor, 8 ALL) on a twice daily x 5 and once daily x 2 schedule (for the ALL models twice daily x 5) for an intended 28 days at a dose of 0.33 mg/kg (Smith et al., 2015). Talazoparib significantly delayed the progression of 21 PDXs (18 solid tumor, 3 ALL) relative to control vehicle-treated mice, but of those only 2 PDXs exhibited regressions (one Wilms tumor, KT-10, and one medulloblastoma, BT-45). While the response of BT-45 remains unexplained, molecular analysis of KT-10 revealed a homozygous or hemizygous frameshift mutation in the *PALB2* gene, the wild-type protein product of which binds to both BRCA1 and BRCA2. The same frameshift mutation in *PALB2* has been reported for a patient with Fanconi anemia (Reid et al., 2007), and talazoparib has subsequently been shown to be active for patients with germline *PALB2* mutations (Gruber et al., 2022). The single PPTP/C pediatric model (KT-10) with a homologous recombination deficiency (HRD) mirrors the clinical setting in which genetic loss of *BRCA1/2*, *BARD1*, *PALB2*, and other genes associated with HRD is uncommon in pediatric cancers.

##### Additional targeted agents with limited activity.

Multiple other targeted agents were tested that showed limited activity, including the PI3K pathway targeted agents described later. For example, the PIM1/2/3 kinase inhibitor SGI-1776 demonstrated some ability to delay tumor progression without inducing regressions (Batra et al., 2012). This suggests a limited role for PIM kinase as a driver for growth and survival of the broad range of pediatric tumor models studied. Similarly, the dual SYK/FLT3 inhibitor TAK-659, which was tested against a small panel of ALL PDX models selected based on high *SYK* and/or *FLT3* mRNA levels and/or the presence of mutated *FLT3*, exhibited low to moderate single-agent activity (Hughes et al., 2023).

#### 3.3.6. JAK1/2 inhibitors

AZD1480 was tested against a total of 52 PDX models (41 solid tumor and 11 ALL, including 7 harboring *JAK1/2* mutations) (Houghton et al., 2014; Suryani et al., 2015), while ruxolitinib was tested against 8 ALL models, both agents being administered on protracted dosing schedules. AZD1480 elicited significant progression delays relative to control vehicle-treated mice in 34 PDXs (32 solid tumor, two ALL) but only induced regressions in two Wilms tumor and one neuroblastoma PDX model. Ruxolitinib was also unable to elicit regressions in any of the ALL PDX models. The limited activity of AZD1480 and ruxolitinib for the ALL PDXs tested was despite the models being highly represented by Ph-like and ETP subtypes harboring *JAK1* or *JAK2* mutations. This suggests that the level of sensitivity to kinase inhibition that is conferred by gene fusions like *BCR::ABL1* for Ph^+^ ALL may not be conferred by point mutations in *JAK1* and *JAK2* for Ph-like ALL. Alternatively, successful treatment may require deeper and more prolonged JAK inhibition, as suggested by genetic modeling of *JAK2*-driven myeloproliferative neoplasms (Dunbar et al., 2024).

The experience described above documents that the PPTP/PPTC tested a broad range of agents that target 1) specific protein kinases, 2) the protein products of mutated genes and gene fusions, and 3) proteins in a synthetic lethal relationship with a mutated gene. For these agents, tumor regressions were rarely observed unless a PDX model expressed a genomic alteration specifically associated with sensitivity to that targeted agent. These observations provide a cautionary note to those attempting to extend the spectrum of patients that can be treated with a targeted agent beyond the genomically-defined population that is relevant to the agent’s target.

### 3.4. Antibody drug conjugates (ADCs) are often effective in preclinical models, but overprediction of activity occurs

Over the past decade, there has been a marked increase in the number of ADCs entering clinical evaluation, and as of January 2024, there were 13 ADCs with regulatory approval for oncology indications and close to ten times this number in clinical trials (Ma, Durga, Wang, Yao, & Wang, 2024). The ability of ADCs to robustly induce tumor regressions in tumors overexpressing their target antigen makes them among the most promising drug classes for pediatric oncology. To date, however, only two ADCs, brentuximab vedotin (targeting CD30 (TNFRSF8)) and inotuzumab ozogamicin (targeting CD22), have shown convincing utility for pediatric patients (Castellino et al., 2022; Lowe et al., 2021; O’Brien et al., 2022; Pennesi et al., 2022), and ADCs for pediatric solid tumors have shown limited activity. A challenge for utilizing ADCs for pediatric solid tumors is that there are few solid tumor correlates of the surface antigens like CD19, CD22, and CD30 (TNFRSF8) that show high expression within a restricted set of normal cell types and that are shared between pediatric and adult leukemias and lymphomas. The high priority pediatric solid tumor antigens discussed subsequently (*e.g.*, oncofetal oncoproteins) are in general not shared with common adult cancers. The PPTP/C has tested multiple ADCs, with most demonstrating considerable activity directly related to documented expression levels of the target protein. ADCs have been among the most active agent classes studied by the PPTP/C, with an ORR of 59 % for both solid tumor and ALL models across all ADCs studied (Table 5). ADCs tested by the PPTP/C included agents targeting CD19 (Carol et al., 2013; Jones et al., 2019), CD56 (NCAM1) (Wood et al., 2013), CD123 (IL3RA) (Evans et al., 2019), B7-H3 (CD276) (Kendsersky et al., 2021), DLL3 (Krytska et al., 2022), DLK1 (Weiner et al., 2024), GPNMB (Kolb et al., 2014), HER2 (ERBB2) (Hingorani et al., 2022), LRRC15 (Hingorani et al., 2021), and ROR1 (Lock et al., 2021).

Most of the ADCs studied by the PPTP/C have not had corresponding evaluations in pediatric clinical trials. For two ADCs (glembatumumab vedotin targeting GPNMB and lorvotuzumab mertansine targeting CD56) for which PPTP/C testing and pediatric phase 2 clinical trials have each occurred, the PPTP/C results over-predicted for clinical activity for the ADCs. Glembatumumab vedotin demonstrated maintained complete response (MCR) in three of six osteosarcoma models, growth delay in two models and no growth delay in one model (Kolb et al., 2014). However, in a phase 2 clinical trial with 22 recurrent osteosarcoma patients, only one patient had a partial response, and two had stable disease (Kopp et al., 2019). Similarly, for lorvotuzumab mertansine objective responses were observed in 9 of 25 (38 %) models including, 3 of 7 neuroblastoma xenografts, and 2 of 7 rhabdomyosarcoma xenografts (Wood et al., 2013). By contrast, the clinical trial for lorvotuzumab mertansine enrolled 12 neuroblastoma and 17 rhabdomyosarcoma patients and among these a single rhabdomyosarcoma patient had a partial response (Geller et al., 2020).

Although it is difficult to precisely determine the reasons for overprediction of activity for these two agents, several factors may have contributed. For lorvotuzumab mertansine for neuroblastoma, one factor may be its tubulin-binding payload, as drug transporters in neuroblastoma limit the activity of cytotoxic agents in this drug class (Buongervino et al., 2021). Another potential factor is the doses of the agents used for preclinical testing in comparison to those administered to patients. de Goeij and Lambert noted that the volume of plasma per kg of body weight is very similar in mice and humans (approximately 40 ml/kg) (Davies & Morris, 1993), and that therapeutic activity should be observed at comparable dose levels in both species assuming that the pharmacokinetic properties are reasonably similar (de Goeij & Lambert, 2016). The body-weight adjusted dose per 3–4 week treatment course for FDA-approved ADCs varies by the type of payload utilized: 1.8 to 3.75 mg/kg for vedotin (Monomethyl auristatin E, MMAE) and mafodotin (Monomethyl auristatin F, MMAF), 3.6 mg/kg for emtansine (DM4), 0.05 to 0.4 mg/kg for ozogamicin (calicheamicin), 5.4 mg/kg for deruxtecan, 20 mg/kg for govitecan (SN-38) and 0.15 mg/kg for (tesirine pyrrolobenzodiazepine dimer) (Lee, 2021; Liao et al., 2021). For lorvotuzumab mertansine, the PPTP/C used a 15 mg/kg weekly x 3 schedule, a dose per course that is approximately 7-fold greater than the dose per course used in the pediatric clinical trial (Geller et al., 2020; Wood et al., 2013). For glembatumumab vedotin, the PPTP/C used a dose of 2.5 mg/kg weekly x 3, which was 4-fold higher than the dose used for clinical testing (Kolb et al., 2014; Kopp et al., 2019).

Another factor that may lead to differences in preclinical and clinical estimates of activity is differences in ADC clearance between species. For example, the antibodies used in ADCs may not be cross reactive with the corresponding murine antigen, potentially leading to increased tolerability due to reduced on-target effects in mice compared to patients and increased clearance in patients compared to mice. ADC metabolism can also differ between species, as illustrated by murine, but not human, plasma carboxylesterase 1c (CES1c) being able to cleave ADCs with some valine-citrulline linkers leading to extracellular release of the payload and markedly reduced half-life in mice following *in vivo* administration (Anami et al., 2018; Dorywalska et al., 2016; Ubink et al., 2018). Dissociation of drug from the antibody in murine models can lead to reduced on-target ADC activity as well as ADC effects that are independent of antibody binding.

Going forward, steps that may improve the success rate for translating preclinical activity to clinical activity for ADCs include: 1) utilizing doses and schedules for preclinical testing that align with those used in clinical trials; 2) evaluating systemic exposures in preclinical models to confirm that drug exposures are comparable to those achieved in patients; 3) evaluating “control” ADCs that utilize the same linker and payload attached to an isotype-matched, non-reactive antibody to confirm dependence of activity on target antigen expression; and 4) focusing on surface antigens that are expressed at high rates on relevant pediatric cancers and not on normal childhood tissues. Surface antigens that are a high priority for pediatric cancers include CD276 (B7-H3) (osteosarcoma, Wilms tumor, neuroblastoma, and others) (Gorlick et al., 2024; Kendsersky et al., 2021; Majzner et al., 2019), ALK (neuroblastoma and fusion-positive rhabdomyosarcoma) (Sano et al., 2019), ERBB3 (HER3) (fusion-positive rhabdomyosarcoma and hepatoblastoma) (Kurmasheva et al., 2024), GPC2 (neuroblastoma) (Bosse et al., 2017; Raman et al., 2021), GPC3 (hepatoblastoma and Wilms tumor), GD2 sialic acid-containing glycosphingolipid (neuroblastoma) (Amendt et al., 2024), IL1RAP, ENPP1, and STEAP1 (Ewing sarcoma) (Mooney et al., 2024; Zhang et al., 2021), LRRC15 (osteosarcoma) (Demetri et al., 2021; Hingorani et al., 2021; Slemmons, Mukherjee, Meltzer, Purcell, & Helman, 2021), and others (Wang et al., 2022). As well, the heterogeneity of expression of some ADC targets within a specific cancer type may require a diagnostic test to identify patients most likely to benefit from treatment with the ADC being considered. For some of the surface antigens listed above that are a priority for pediatric cancers, much of the work to date has been with academic-developed tool compound ADCs. Defining a clinical development path for these agents is a pressing unmet need for the pediatric oncology community.

### 3.5. PDX models overpredict for activity of mitotic kinase inhibitors, which is likely a result of faster cell cycling times in PDX models compared to patient tumors

The PPTP/C evaluated four mitotic kinase inhibitors: the Aurora kinase A (AURKA) inhibitor alisertib (MLN8237) (Carol et al., 2011; Maris et al., 2010; Mosse et al., 2019), the CENPE inhibitor GSK923295A (Lock et al., 2012), the PLK1 inhibitor volasertib (BI6727) (Gorlick et al., 2014), and the KSP (KIF11) inhibitor ispinesib (SB-715992) (Carol et al., 2009). The ORR across all four agents was 23 % ORR for solid tumor models and 79 % ORR for ALL models (Table 6). Among solid tumor models, the lowest ORR was for osteosarcoma (6 %). Despite the preclinical activity signals, only limited activity has been observed in the clinic, with dosing of agents in this class limited by on-target bone marrow and gastrointestinal toxicity. No mitotic kinase inhibitors have advanced to regulatory approval for a cancer indication. Several of these agents have proceeded to clinical testing in children with cancer, including alisertib (DuBois et al., 2016; DuBois et al., 2018; Mosse et al., 2012), ispinesib (Souid et al., 2010), and volasertib (Doz et al., 2019); the number of objective responses in the pediatric trials was very low and myelosuppression was common. Hence, the mitotic kinase inhibitors represent a class of agents for which the PPTP/C testing provided activity signals, but for which testing in children failed to identify clinical activity. False positive signals such as these are important to analyze so that future testing projects can be designed to avoid over-predicting for clinical activity so that negative phase 2 clinical trials such as that for alisertib can be avoided (Mosse et al., 2019).

One issue in understanding the failure to translate preclinical activity for the mitotic kinase inhibitors into clinical activity is the essential nature of these kinases to both cancer cells and to normal tissues (Komlodi-Pasztor, Sackett, & Fojo, 2012; Yan et al., 2020). The genes encoding the mitotic kinases studied by the PPTP/C are scored as common essentials by CRISPR drop-out screening in the DepMap resource (Tsherniak et al., 2017), suggesting that they are essential to the maintenance of normal proliferating tissues. The faster turnover and proliferation for some normal tissues (*e.g.*, bone marrow and gut cells) in comparison to proliferation and growth in most tumors provides a cautionary note for the likelihood for therapeutic window for mitotic kinase inhibitors (Richardson, Allan, & Le, 2014; Tubiana, 1989). Another factor explaining the overprediction by preclinical models is that these models proliferate at faster rates than tumors in patients (Komlodi-Pasztor et al., 2012). In patients, the fastest growing tumors may double every three weeks (Tubiana, 1989), while many of the preclinical models studied by the PPTP/C will quadruple in volume within two weeks. The lower response rates of osteosarcoma models to the mitotic kinase inhibitors compared to response rates for other solid tumor models supports the relationship between proliferation rate and response to mitotic kinase inhibitors, as the osteosarcoma panel has the slowest growth rates among the PPTC/P solid tumor panels. Another measure of the faster proliferation rate for the preclinical models is that many cell cycle genes are expressed at 3-to 5-fold higher levels in the PPTP/C preclinical models in comparison to clinical specimens for the same diagnoses (Neale et al., 2008). The higher cell cycling rates for preclinical models compared to tumors in patients means that a higher percentage of tumor cells will be susceptible to mitotic kinase inhibitors at each treatment in comparison to the percentage that will be susceptible in patients and that there will be a higher likelihood of a therapeutic response in the preclinical models.

One lesson that can be learned from the PPTP/C and from the experience of other research teams studying mitotic kinase inhibitors is that preclinical models like those used by the PPTP/C will inherently overpredict the activity of agents that are active against cells only during mitosis. This overprediction needs to be factored into any analysis of the preclinical testing results for such agents. Another lesson is that overprediction may be reduced by using clinically relevant doses and schedules, as illustrated by experience with alisertib. A schedule that utilized more continuous dosing of alisertib showed greater preclinical activity in ALL models than a schedule that used more intermittent dosing (mimicking the clinical setting in which intermittent dosing followed by time for recovery from myelosuppression and other toxicities is required) (Mosse et al., 2019). The intermittent schedule that better approximated that used in the clinic showed limited activity for alisertib against ALL models that was more akin to that observed in patients.

### 3.6. BCL2 and MDM2 inhibitors are rarely effective in solid tumor models as single agents

The development of small molecule inhibitors of BCL2 family anti-apoptotic proteins resulted in a paradigm shift for the treatment of hematologic malignancies such as chronic lymphocytic leukemia (CLL) and acute myeloid leukemia (AML) (Roberts, 2020). Moreover, drugs that reactivate wild-type TP53 by inhibiting its interaction with human/mouse double minute 2 (MDM2/*Mdm2* inhibitors), thereby inducing tumor cell apoptosis, have received considerable attention over the past two decades as a novel treatment strategy for both solid and hematolymphoid tumors (Konopleva et al., 2020). Therefore, it was of interest for the PPTP/C to test the *in vivo* single agent activity of several BCL-2 family inhibitors and MDM2 inhibitors across its diverse panels of pediatric cancer PDX models. Accordingly, the *in vivo* activity of the BCL2/BCL-xL (BCL2L1)/BCL-W (BCL2L2) inhibitor navitoclax (ABT-263) (Lock et al., 2008; Suryani et al., 2014), the BCL2 inhibitor venetoclax (ABT-199) (Khaw et al., 2016), the MCL1 inhibitor AMG 176 (Lock et al., 2022), the MDM2 inhibitors RG7112 (Carol et al., 2013; Richmond et al., 2015) and MK-8242 (Kang et al., 2016) and the MDM2 inhibitor/DNA-damaging agent serdemetan (JNJ-26854165) were evaluated (Smith et al., 2012).

#### 3.6.1. BCL-2 family inhibitors

When navitoclax was administered orally at a dose of 100 mg/kg daily for 21 days, 35 of 36 solid tumor PDX models showed progressive disease (Lock et al., 2008). In contrast, when tested using the same regimen against pediatric ALL PDX models, navitoclax induced objective responses in 4 of 8 models at initial testing and in expanded testing induced objective responses in 19 of 31 (61 %) PDX models (Lock et al., 2008; Suryani et al., 2014). Due to the disappointing results with navitoclax against the pediatric solid tumor PDXs, venetoclax was only tested against the pediatric ALL PDX models using the same regimen as was used for navitoclax. Venetoclax induced objective responses in 5 of 19 (26 %) PDX models (Khaw et al., 2016), which represented a marked reduction in activity compared with navitoclax despite both agents being well tolerated. The notable decrease in *in vivo* activity of venetoclax compared with navitoclax against the pediatric ALL PDX models suggests a reduced dependency of pediatric ALL on BCL2 compared with, for example, CLL (Ashkenazi, Fairbrother, Leverson, & Souers, 2017; Roberts, 2020). The exception to greater activity for navitoclax among the ALL models was the *KMT2A*-rearranged models for which venetoclax and navitoclax had comparable *in vivo* activity (Khaw et al., 2016). The PPTP/C *in vivo* results for venetoclax are supported by the observation that the *KMT2A*::*AFF1* fusion upregulates *BCL2* gene expression (Benito et al., 2015; Godfrey et al., 2017) and that *in vitro* sensitivity to venetoclax for *KMT2A*-rearranged models, but not for other ALL models, was highly correlated with that of navitoclax (Khaw et al., 2016). The MCL1 inhibitor AMG 176 induced no objective responses among 37 ALL models tested (Lock et al., 2022).

The marked difference in navitoclax activity between pediatric solid tumor and ALL PDX models suggests a profound difference in their dependency on the anti-apoptotic proteins BCL2, BCL-xL and/or BCL –W. This hypothesis has been difficult to test in the clinical setting, since the major dose-limiting toxicity of thrombocytopenia has resulted in limited clinical evaluations of navitoclax (Ashkenazi et al., 2017), and it has not achieved regulatory approval for any indication. In a limited number of reports of navitoclax in adult cancer clinical trials, it appeared to exhibit greater activity against lymphoid malignancies than solid tumors (de Vos et al., 2021; Tolcher et al., 2015), but there is no single agent data for navitoclax in children with leukemia.

Venetoclax is approved in combination with either azacitidine, decitabine, or low dose cytarabine for elderly adults with newly diagnosed AML who cannot be treated with intensive chemotherapy. Venetoclax is also approved for CLL and small lymphocytic lymphoma (SLL) in adults. Clinical experience for venetoclax in children has primarily been for AML in combination with standard-of-care agents (Karol et al., 2020). For ALL, a retrospective study described the use of venetoclax in combination with standard-of-care agents (Gibson et al., 2022). The contribution of venetoclax to the reported outcome of patients described in these reports cannot be isolated. Similarly, a phase 1 study of venetoclax with low dose navitoclax and chemotherapy was conducted in pediatric and adult patients with relapsed/refractory (R/R) ALL or lymphoblastic lymphoma, and although the results were considered promising, the contribution of neither venetoclax nor navitoclax could be ascertained (Pullarkat et al., 2021). There remains interest in evaluating venetoclax for infants with *KMT2A*-rearranged ALL (Ibrahimova, Pommert, & Breese, 2021), and a Children’s Oncology Group clinical trial (NCT06317662) is evaluating the addition of venetoclax to a standard chemotherapy regimen used to treat infants with this ALL subtype.

#### 3.6.2. MDM2 inhibitors

The *cis*-imidazoline (Nutlin) derivative RG7112 was administered orally at a dose of 100 mg/kg for 14 days and induced objective responses in 5 of 29 (17 %) solid tumor PDX models. Similarly, the MDM2 inhibitor MK-8242, which was administered orally at a dose of 125 mg/kg on days 1–5 and 15–19, induced objective responses in 19 % of solid tumor models (Kang et al., 2016). Neither of the MDM2 inhibitors induced objective responses in osteosarcoma or neuroblastoma models, but in the limited number of models tested objective responses were noted for Ewing sarcoma (25 %), rhabdomyosarcoma (14 %), medulloblastoma (1 of 2), and other histologies.

The MDM2 inhibitors showed higher activity against ALL models compared to solid tumor models. RG7112 elicited objective responses in 13 of 15 (87 %) PDX models, including 7 of 7 infant *KMT2A*r ALL models (Carol, Reynolds, et al., 2013; Richmond et al., 2015). Due to tolerability issues in NOD/SCID mice the MK-8242 dose was reduced to 75 mg/kg for the ALL efficacy study, but was still able to induce objective responses in 8 of 9 models (89 %), albeit with 6 of the responses being PRs and only 2 being CRs (Kang et al., 2016).

Translation of preclinical results to clinical success for MDM2 inhibitors has been unsuccessful to date for adult cancers. The main challenge for MDM2 inhibitors has been their on-target hematopoietic and gastrointestinal toxicity, with these limiting both the dose and duration of treatment (Erba et al., 2019; Ravandi et al., 2016; Ray-Coquard et al., 2012; Stein et al., 2021). MDM2 inhibition with concomitant TP53 activation induces cytotoxicity in hematopoietic progenitors and leads to deleterious effects on erythroid and megakaryocyte differentiation (Iancu-Rubin et al., 2014; Mahfoudhi et al., 2016). Delayed onset thrombocytopenia resulting from effects of MDM2 inhibition on platelet progenitors is characteristic of MDM2 inhibitors (Bauer et al., 2021; Gluck et al., 2020; Patnaik et al., 2015). RG7112 was evaluated in adults with liposarcoma, a cancer that commonly shows MDM2 amplification, and it failed to show tumor-regressing activity (Ray-Coquard et al., 2012). Low objective response rates for patients with solid tumors have also been reported for other MDM2 inhibitors (Bauer et al., 2021; de Jonge et al., 2017; Stein et al., 2021; Wagner et al., 2017), while response rates for hematological malignancies like AML have been modestly higher for some agents studied (Erba et al., 2019; Konopleva et al., 2020; Stein et al., 2021). However, a phase 3 trial evaluating the addition of idasanutlin to cytarabine in adults with relapsed/refractory AML was stopped for futility based on efficacy results at an interim analysis (Konopleva et al., 2022). The MDM2 inhibitor brigimadlin remains under clinical evaluation and has shown objective responses for patients with MDM2-amplified well-differentiated liposarcoma as well as prolonged stable disease for patients with dedifferentiated liposarcoma (LoRusso et al., 2023). Pediatric experience with MDM2 inhibitors is limited, and a clinical trial evaluating idasanutlin (NCT04029688) is now closed to accrual but results are not yet available. The on-target toxicity observed for this class of agents in adult cancer patients raises concerns about the likelihood of identifying a pediatric population for which a favorable therapeutic window exists.

### 3.7. PI3K pathway inhibitors rarely induce objective responses in solid tumor models

Activation of the PI3K signaling pathway in pediatric cancer has been reported for both hematologic (Min et al., 2003; Steelman et al., 2004) and solid tumors [reviewed in (Loh et al., 2013)]. Reports of PI3K/mTOR activation in multiple childhood cancers led the PPTP/C to evaluate 9 inhibitors in this pathway against both solid tumors and ALL, including: PI3K inhibitors (pilaralisib [SAR245408, XL147] (Reynolds et al., 2013), voxtalisib [XL765], copanlisib and duvelisib), AKT inhibitors [GSK690693 (Carol et al., 2010) and MK2206 (Gorlick et al., 2012)]; mTORC1/2 inhibitors [AZD8055 (Houghton et al., 2012) and sapanisertib (MLN0128, TAK-228) (Kang et al., 2014)]; and a mTORC1-specific inhibitor [rapamycin (Houghton et al., 2008)].

Among the five PI3K inhibitors studied by the PPTP/C, duvelisib and acalisib show specificity for PI3Kδ, while copanlisib and pilaralisib are considered to have pan-PI3K inhibitory activity, and voxtalisib inhibits both PI3K and mTOR. PI3Kδ is primarily restricted to hematopoietic cells and plays a signaling role in multiple pathways relevant to mature B-cell malignancies. PI3Kδ inhibitors are active against CLL, and duvelisib as well as idelalisib are approved by FDA for the treatment of CLL (Miller et al., 2015; Patel, Danilov, & Pagel, 2019). Given the hematopoietic specificity of PI3Kδ expression, duvelisib was only studied in ALL models and was evaluated in these models using the single mouse trial (SMT) design (Randall et al., 2023). Among 29 ALL models evaluated, only one BCP-ALL model had a PR and the remaining models had PD responses (Table 7). This suggests that the utility of PI3Kδ inhibitors for adults with CLL does not translate to pediatric ALL. Consistent with this conclusion, PPTP/C testing of the pan-PI3K inhibitor pilaralisib (SAR245408) did not show objective responses in 7 ALL models (Table 7) (Reynolds et al., 2013).

For solid tumor models, including brain tumors, the pan-PI3K inhibitor pilaralisib was tested against 38 models of multiple histologies, the PI3K and mTOR inhibitor voxtalisib was tested against 2 GBM models, and the pan-PI3K inhibitor copanlisib was tested against 6 osteosarcoma models (Reynolds et al., 2013). No objective responses were observed for any of these agents, and only 5 of 46 (11 %) models achieved a PD2 response (Table 7). The only PI3K inhibitor approved for the treatment of non-hematopoietic cancers in adults is alpelisib, which shows relative specificity for PI3Kα and which is approved for a subset of advanced or metastatic breast cancer patients with *PIK3CA* mutations whose disease has progressed on or after an endocrine-based regimen (Narayan et al., 2020). Consistent with the PPTP/C testing results for PI3K inhibitors in solid tumor models, alpelisib was not effective for patients with breast cancer whose tumors were *PIK3CA* wild-type. Activating *PIK3CA* mutations are exceedingly uncommon among pediatric cancers (< 1 % of leukemias and solid tumors and < 3 % of brain tumors), and the PPTC panel of approximately 240 models has none with activating *PIK3CA* mutations (St. Jude Cloud PeCan, 2022). The totality of evidence indicates a limited role for PI3K inhibitors for pediatric cancers.

Two AKT inhibitors were evaluated by the PPTP/C: GSK690693 which is an ATP-competitive inhibitor (Rhodes et al., 2008), and MK-2206 which is an allosteric AKT inhibitor (Hirai et al., 2010). Together GSK690693 and MK-2206 were studied against 41 and 30 solid tumor models, respectively, as well as against 8 ALL models (Table 7) (Carol et al., 2010; Gorlick et al., 2012). Only one PR was observed for either agent for solid tumor and for or ALL models, and PD2 responses occurred in only 4 % and 25 % of solid tumor and ALL models, respectively. Clinical development for both MK-2206 and GSK690693 has been halted. MK-2206 showed limited activity in single agent trials and did not appear to provide benefit when used in combination (Coleman, Moyers, Harbery, Vivanco, & Yap, 2021). GSK690693 was stopped after its phase 1 evaluation, reportedly because of safety concerns related to hyperglycemia (Nitulescu et al., 2016). The only AKT inhibitor approved by FDA for a cancer indication is capivasertib. Capivasertib induced objective responses as a single agent in 20 % to 30 % of patients whose cancer had an *AKT1* E17K mutation (Hyman et al., 2017; Kalinsky et al., 2021; Smyth et al., 2020), a mutation that is exceptionally uncommon in pediatric cancers (St. Jude Cloud PeCan, 2022). Capivasertib is approved for use in combination with fulvestrant for patients with advanced breast cancer whose tumors have one or more *PIK3CA*/*AKT1*/ *PTEN* alterations (Nierengarten, 2024). Based on the absence of activity for AKT inhibitors in the PPTP/C models, the narrow range of adult cancer populations in which AKT inhibitors appear to have efficacy, and the low frequency of the primary predictive genomic biomarker in the pediatric population, there is limited rationale for developing an AKT inhibitor for pediatric cancers.

The macrocyclic lactone rapamycin (sirolimus) is an allosteric inhibitor specific for inhibition of the mTOR complex 1 (mTORC1). In contrast to other PI3K pathway inhibitors studied by the PPTP/C, rapamycin induced objective responses, with a 14 % ORR among 37 solid tumor models studied and a 38 % ORR among 8 ALL models studied (Table 7). An additional 30 % of solid tumor models had a PD2 response. Objective responses were observed for several histologies, including malignant rhabdoid tumor, rhabdomyosarcoma, and osteosarcoma. Both T-ALL xenografts against which rapamycin was tested had objective responses (Houghton, Morton, Kolb, Gorlick, et al., 2008). Rapamycin was also studied in selected solid tumor models in combination with either cyclophosphamide or vincristine, and the combination was significantly more effective than the respective standard agents used alone at their maximum tolerated doses (MTD) for most evaluable models (Houghton et al., 2010). The combination of rapamycin with the anti-IGF1 receptor (IGF1R) MAB cixutumumab (IMC-A12) demonstrated therapeutic enhancement in some sarcoma models (Kolb et al., 2012). Based on PPTP/C results, a randomized phase 2 trial was conducted for patients with relapsed rhabdomyosarcoma comparing the mTOR inhibitor temsirolimus to bevacizumab, with each agent being given with vinorelbine plus cyclophosphamide (Mascarenhas et al., 2019b). Patients on the temsirolimus arm had significantly prolonged EFS compared to those on the bevacizumab arm. Building upon the results from this randomized phase 2 study, a phase 3 study was developed to evaluate the addition of temsirolimus to standard therapy in newly diagnosed patients with intermediate-risk rhabdomyosarcoma (NCT02567435). As discussed further in the combination testing section, the addition of temsirolimus to standard therapy did not lead to improved outcome for patients with intermediate-risk rhabdomyosarcoma (Gupta et al., 2024).

Considering the activity of rapamycin against pediatric preclinical models, it was anticipated that small molecule ATP-competitive inhibitors of mTOR kinase that inhibited both mTORC1 and mTORC2 would have greater antitumor activity. Two TOR kinase inhibitors, AZD8055 and sapanisertib (MLN0128, TAK-228) were evaluated in 38 and 31 solid tumor models, respectively, and in 7 ALL models, (Table 7) (Houghton et al., 2012; Kang et al., 2014). No objective responses were observed for either solid tumor or ALL models, and for the solid tumor models PD2 or stable disease (SD) responses were observed in only 19 % of models tested. Clinical development of AZD8055 in adults did not advance past early phase clinical trial testing. Sapanisertib continues in limited clinical development, primarily focusing on patients with squamous non-small cell lung cancer with *KEAP1/NRF2 (NFE2L2*) mutations (Calithera Biosciences, 2021; Paik et al., 2023). Evaluations of sapanisertib against other cancer types (*e.g.*, sarcomas, renal cell carcinoma, ALL, prostate cancer, and breast cancer) showed limited evidence of clinical activity, either as a single agent or in combination (Al-Kali et al., 2019; Garcia-Saenz et al., 2022; Graham et al., 2018; Ingham et al., 2020; McGregor et al., 2022).

To summarize, targeting the PI3K/mTOR pathway produced modest to minimal antitumor activity in the pediatric preclinical models evaluated. Rapamycin, alone or in combination, showed the greatest activity, which was largely restricted to sarcomas. Small molecule inhibitors of PI3K, AKT or TOR kinases showed a disappointing lack of antitumor activity against pediatric preclinical models. In retrospect, the limited pediatric preclinical activity is not surprising as experience for adult cancers has shown that genomic alterations leading to PI3K pathway activation are the primary predictors of clinical activity for pathway inhibitors, as illustrated by the approval of the PIK3CA inhibitor alpelisib for use in combination with fulvestrant for patients with advanced or metastatic breast cancer whose tumors having activating *PIK3CA* mutations (Narayan et al., 2021). Likewise, the AKT inhibitor capivasertib was approved in combination with fulvestrant for patients with advanced breast cancer whose tumors have one or more *PIK3CA*/*AKT1*/ *PTEN* alterations (Nierengarten, 2024), and the mTOR inhibitor everolimus is approved for patients with conditions associated tuberous sclerosis complex with *TSC1* or *TSC2* mutations that lead to mTOR activation (Previtali et al., 2023). For pediatric cancers, data from St Jude Children’s Research Hospital PeCan genomics data portal show that only 1 % of pediatric cancers have activating *PIK3CA* mutations, with pediatric high-grade gliomas being the only diagnosis with 10 % or higher *PIK3CA* mutation frequency (McLeod et al., 2021). *AKT1* mutations are much less common than *PIK3CA* mutations among childhood cancers (McLeod et al., 2021).

### 3.8. Preclinical testing of anti-IGF1 receptor (IGF1R) MABs illustrates the importance of testing multiple PDX models for a target histology

IGF1R is broadly expressed in a number of pediatric solid tumors, and biological rationale existed for considering it a priority therapeutic target for cancers like Ewing sarcoma (Kurmasheva et al., 2009; Kurmasheva & Houghton, 2006; Scotlandi et al., 1998; Scotlandi et al., 2002), rhabdomyosarcoma (Bid, Zhan, Phelps, Kurmasheva, & Houghton, 2012; Thimmaiah et al., 2003; Zhan, Shapiro, & Helman, 1994), neuroblastoma (El-Badry et al., 1991; Misawa et al., 2000), and other childhood cancers. The ligands for IGF1R (IGF1 and IGF2) are differentially expressed across pediatric cancers. Within the PPTP/C *in vivo* models, rhabdomyosarcoma and Wilms tumor models consistently show very high levels of IGF2 expression and low levels of IGF1 expression, while IGF2 expression is lower and more variable for neuroblastoma and osteosarcoma models and is lower still for Ewing sarcoma. Expression of IGF1R is highest for the rhabdomyosarcoma and Wilms tumor models, and lower and more variable for osteosarcoma, neuroblastoma, and Ewing sarcoma models.

The PPTP/C studied two IGF1R-targeting antibodies, robatumumab (SCH 717454) and cixutumumab (IMC-A12) (Houghton et al., 2010; Kolb et al., 2008a). For robatumumab and cixutumumab, the overall ORR among 70 solid tumor models was 6 % (Table 8). Response rates by histology were 18 % for osteosarcoma (2 of 11), 10 % for Ewing sarcoma (1 of 10), and 10 % for rhabdomyosarcoma (1 of 10). No objective responses were observed for neuroblastoma (*n* = 11) or for Wilms tumor (*n* = 6). Substantial slowing of tumor growth (PD2 responses) was observed in 29 % of solid tumor models, with the rate of PD2 responses being highest (60 %) for rhabdomyosarcoma models. For the ALL models tested (*n* = 8) tested against robatumumab, no objective responses were noted.

The PPTP/C also studied BMS-754807, a small molecule inhibitor of IGF1R and the insulin receptor (INSR) (Kolb et al., 2011). BMS-754807 shows equipotent inhibition of IGF1R and insulin receptor (INSR) (Carboni et al., 2009), but inhibition of other kinases may also contribute to the *in vitro* and *in vivo* activity of BMS-754807 (Anastassiadis et al., 2013; Carboni et al., 2009). Only one objective response to BMS-754807 treatment was observed among 37 solid tumor models and there were no objective responses among 8 ALL models (Kolb et al., 2011).

PPTP/C results for cixutumumab were also used to identify mechanisms of resistance to this agent and to better understand why most models do not respond to inhibition of IGF1R signaling. This work built on previous work documenting that insulin receptor (INSR) may play a role in resistance to IGF1R inhibition (Vella, Milluzzo, Scalisi, Vigneri, & Sciacca, 2018). The INSR isoform IR-A differs from the isoform IR-B by lacking exon 11 and is 12 amino acids shorter than IR-B. IR-A shows comparable binding to both insulin and IGF2, while IR-B favors insulin over IGF2. As many pediatric tumors express high levels of IGF2, signaling through IR in response to IGF2 may minimize the impact of blocking IGF1R signaling. Garofalo, et al., showed that for Ewing sarcoma resistance to anti-IGF1R therapy can result from a switch from IGF1/IGF1R to IGF2/IR-A dependency that allows maintained signaling to support proliferation and survival (Garofalo et al., 2011). For the PPTP/C models, higher INSR expression was associated with reduced activity of cixutumumab, consistent with results for adult cancer models showing that signaling through both IR-A and IR-B can mediate resistance to antibodies blocking IGF1R signaling (Forest et al., 2015). Rhabdomyosarcoma and Ewing sarcoma cell lines used by the PPTPC/C were employed to develop evidence for an alternative mechanism of resistance to anti-IGF1R MABs, that being that both intrinsic and acquired resistance are a consequence of redundant signaling by other receptor tyrosine kinases (Shackleford et al., 2023).

Multiple anti-IGF1R MABs entered clinical evaluation, with many of the agents being studied against pediatric cancers (Qu et al., 2017). A remarkable complete response that persisted for over two years in a patient with multiply relapsed Ewing sarcoma enrolled on a phase 1 trial of ganitumab generated hope that this class of agents would find a role in the treatment of Ewing sarcoma (Tolcher et al., 2009). However, as more patients with Ewing sarcoma were enrolled onto clinical trials, it became clear that objective response rates for anti-IGF1R MABs were generally in the 10 % to 15 % range for Ewing sarcoma and that most responses were not prolonged (Juergens et al., 2011; Malempati et al., 2012; Pappo et al., 2011; Schoffski et al., 2013; Tap et al., 2012). For rhabdomyosarcoma (Bagatell et al., 2011; Schoffski et al., 2013), osteosarcoma (Anderson et al., 2016; Bagatell et al., 2011; Weigel et al., 2014), and neuroblastoma (Weigel et al., 2014), responses to anti-IGF1R MABs have been uncommon, although prolonged stable disease has been observed in some patients. Hence, the PPTP/C results for anti-IGF1R MABs have generally matched clinical observations for these agents with objective responses being rarely observed across a range of pediatric solid tumors. Anti-IGF1R MABs have not achieved regulatory approval for any malignant diseases, and their only current clinical indication is for the treatment of thyroid-associated ophthalmopathy [also known as Graves’ ophthalmopathy/orbitopathy or thyroid eye disease] for which teprotumumab (R1507) received FDA approval in 2020 (Douglas et al., 2020; Winn & Kersten, 2021).

## 4. Experimental design considerations and data accessibility

### 4.1. Incorporating human and murine pharmacology into interpretation of preclinical results is critical for successful translation from preclinical testing to clinical trials

The field of oncology drug development is littered with examples of agents that showed promising activity in preclinical mouse models but then went on to fail in the clinic. Consequently, xenograft and genetically engineered mouse models of cancer have been subjected to a good deal of skepticism regarding their ability to predict the clinical activity of novel agents. Historically, extensive preclinical testing carried out by the NCI using conventional xenograft models showed poor predictive value (Hidalgo et al., 2014; Johnson et al., 2001; Peterson & Houghton, 2004). Other studies using PDXs have shown a greater degree of correlation between preclinical and clinical responses for both conventional and novel agents in certain tumor types, including childhood rhabdomyosarcoma (Fiebig et al., 1985; Hidalgo et al., 2014; Houghton, Cook, Lutz, & Houghton, 1984; Houghton, Houghton, & Green, 1982; Peterson & Houghton, 2004).

While the reasons why mouse xenograft models may not reliably predict the clinical activity of a specific drug are likely to be multifactorial, one explanation is the inability or failure to relate systemic drug exposures in mice to those that are achievable in humans. An important illustration of this disparity was the remarkable preclinical activity of irofulven (MGI-114) against pediatric cancer PDXs, albeit at systemic exposures in mice some 6- to 20-fold those achievable in humans [(Eckhardt et al., 2000; Leggas et al., 2002) and reviewed in (Peterson & Houghton, 2004)]. Even accounting for slight differences in protein binding between species, the systemic exposures required to elicit even minimal antitumor activity in mouse xenograft models were around 6-fold higher than those achievable in humans. In contrast, pharmacokinetically guided dosing identified the topoisomerase I poison topotecan to be an active agent in the treatment of neuroblastoma (Santana et al., 2003; Zamboni et al., 1998), supporting the importance of evaluating new agents in preclinical mouse models while maintaining focus on modeling systemic drug exposures between mice and humans. For new agents in early development this may be difficult, since first in human clinical trials are unlikely to have been initiated and drugs are tested at their maximum tolerated dose (MTD) in mice. However, the PPTP/C frequently tested new agents that were advanced in adult cancer clinical trials, which allowed leveraging of available information on optimal scheduling and systemic exposures relevant between mice and humans, several examples of which are discussed below.

#### 4.1.1. The aurora A kinase inhibitor alisertib (MLN8237)

Alisertib was originally tested by the PPTP/C on a schedule of twice daily x 5 repeated for an intended 6 weeks for the solid tumor PDXs and 3 weeks for the ALL PDXs based on best available information about clinical development plans (Maris et al., 2010). Remarkable activity was observed, with an ORR of 36 % across 47 solid tumor and ALL PDX models and prolonged regressions elicited in 3 of 7 neuroblastoma PDXs and 6 of 6 ALL PDXs. Consequently, and along with some clinical antitumor activity observed in adult cancer clinical trials, alisertib was prioritized for a Children’s Oncology Group (COG) phase I/II trial in children and adolescents with relapsed or refractory solid tumors evaluated on both a once and twice daily schedule (Mosse et al., 2012). Based on the adult cancer phase I trial a pediatric phase II clinical trial was designed in children with relapsed/refractory solid tumors or leukemia using a once daily x 7 schedule followed by a 14-day break (since a continuous treatment schedule was too myelosuppressive), with overall disappointing objective response rates (Mosse et al., 2019). In an example of “reverse translation”, the PPTP/C subsequently showed that the *in vivo* responses of two of the ALL PDXs included in the original PPTP/C testing of alisertib were markedly attenuated when evaluated using the intermittent daily x 7 schedule *versus* the continuous daily x 5 × 3 weeks schedule originally utilized (Mosse et al., 2019). Despite plasma trough levels of alisertib in children enrolled in the phase II clinical trial reaching sufficient levels for efficacy based on the mouse preclinical data (Carol et al., 2011; Mosse et al., 2019), it is likely that the poor clinical efficacy was due to having to administer alisertib to children on an intermittent dosing schedule because of myelosuppression (Mosse et al., 2019). While the pediatric phase I data were unknown at the time of the PPTP/C testing of alisertib, this example serves to illustrate the importance of modeling mouse *versus* human drug schedules and exposures in preclinical drug testing.

#### 4.1.2. The PLK inhibitor volasertib (BI6727)

The relatively high expression of Polo-like kinase 1 (PLK1), a serine/threonine kinase that regulates progression through mitosis, across multiple pediatric cancer subtypes (Rokita et al., 2019) made it an attractive target for novel therapeutics. The potent PLK1 inhibitor volasertib (BI6727) was tested by the PPTP/C against 40 solid tumor and ALL PDXs weekly for an intended 3 weeks at a dose of 30 mg/kg (solid tumor PDXs) or 15 mg/kg (ALL PDXs). While several PDXs had to be excluded due to drug toxicity, volasertib significantly delayed tumor progression relative to control vehicle-treated mice in >50 % of PDXs, induced objective responses in 5 PDXs (4 solid tumor, one ALL), thereby indicating some potential for clinical activity. However, a comparison of mouse and human systemic exposures revealed that mice tolerate around 10-fold higher volasertib levels than humans (Ottmann et al., 2019; Rudolph et al., 2009) which, when combined with more frequent administration of volasertib in mice, aligns with the poor clinical activity observed in pediatric cancer patients (Doz et al., 2019).

#### 4.1.3. The AKR1C3-activated prodrugs PR-104 and OBI-3424

PR-104 was originally developed as a hypoxia-activated DNA alkylating pre-prodrug for the treatment of hypoxic solid tumors (Patterson et al., 2007). It was tested against the PPTP/C pediatric cancer PDX models on a once weekly schedule x 6 at its MTD of 550 mg/kg (Houghton et al., 2011). Remarkable activity was observed, with objective responses in 23 of 37 solid tumor PDXs and 7 of 7 evaluable ALL PDXs. Of note, no objective responses were observed in 5 of 5 solid tumor PDXs when the PR-104 dose was attenuated to 270 or 110 mg/kg (Houghton et al., 2011). In a parallel clinical trial, the human equivalent achievable dose in mice was determined to be 50–200 mg/kg (Patel et al., 2011), suggesting that the dose of PR-104 that was effective against the PPTP/C PDXs in mice was not achievable in humans. In separate studies it was shown that the prodrug of PR-104, PR-104 A, could be activated under aerobic conditions by the aldo-keto reductase family 1 member C3 (AKR1C3) (Guise et al., 2010), that T-ALL PDXs expressed significantly higher levels of AKR1C3 compared with B-ALL PDXs (Moradi Manesh et al., 2015), and that PR-104 was effective *in vivo* against T-ALL PDXs at a clinically-relevant dose level (Benito et al., 2011; Moradi Manesh et al., 2015). However, a biomarker-driven clinical trial of PR-104 was not carried out and disappointing efficacy was observed in adult relapsed/refractory acute leukemia patients (Konopleva et al., 2015).

OBI-3424 is a next generation prodrug that is specifically activated by AKR1C3 and not hypoxia. The dose of OBI-3424 selected for PPTC evaluation against ALL PDX models was based on allometric scaling of cynomolgus monkey tolerability and mouse/monkey PK data, rather than the mouse MTD, inferring that the dose selected would produce systemic exposures in mice that were achievable in humans (Evans et al., 2019). When tested at a dose of 2.5 mg/kg on a once weekly x 3 schedule, OBI-3424 elicited objective responses in 5/6 T-ALL PDXs, including almost complete clearance of leukemia cells from the mouse bone marrow at Day 28 (14 days after the last OBI-3424 treatment) (Evans et al., 2019). In 2018, OBI-3424 was granted orphan drug designation by the FDA for the treatment of ALL and is currently being evaluated in a SWOG-sponsored phase 2 study in patients with relapsed/refractory T-ALL in which AKR1C3 protein expression is being assessed retrospectively (ClinicalTrials.gov Identifier: NCT04315324).

The above examples illustrate the critical importance of considering achievable systemic exposures in humans compared to those observed in mice when evaluating new agents in preclinical mouse models to prioritize them for clinical trials in children with high-risk malignancies. For agents early in development, there may be limited data in humans on which to design preclinical testing experiments, but once data are available, they can be incorporated into assessments of the likely clinical relevance of the preclinical data. Moreover, caution should be exerted when interpreting the results for an agent that exerts *in vivo* activity over a narrow dose range to account for possible inter-patient variability in the drug’s bioavailability when assessing clinical efficacy.

#### 4.1.4. KSP (KIF11) inhibitor ispinesib

Results for the KSP (KIF11) inhibitor ispinesib provide an additional insight into how preclinical results can help inform prioritization decisions when the pharmacology of the agent is known in humans. Ispinesib was studied in children with recurrent solid tumors using a weekly for 3 weeks repeated every 28-day schedule (Souid et al., 2010). The MTD was 9 mg/m^2^ weekly for 3 weeks on a 28-day cycle with neutropenia being dose limiting, and no objective responses were observed among 24 patients evaluable for response. There was substantial interpatient variation in drug disposition in children with C_max_ reaching 1.5 mM with a rapid T_1/2a_ but long terminal half-life (16–33 h; 9 mg/m^2^ dose) (Souid et al., 2010). The pharmacology in humans would appear to predict responsiveness based on *in vitro* testing results for pediatric cancer cell lines (median IC_50_ 4.1 nM for 96-h exposure) (Carol et al., 2009). However, ispinesib is highly protein bound in murine plasma with free drug estimated at 0.6 % (Gampa et al., 2020), and it has a high level of binding to human albumin (Pasche, Laleu, & Keiser, 2018). As a result, in patients free drug levels are likely above the ispinesib IC_50_ for less than 24 h after each weekly ispinesib infusion. Given the cell-cycle specific activity of ispinesib, the short period of each treatment course during which tumor cells are exposed to effective free drug levels provides an explanation for its limited clinical activity.

It can be challenging to match mouse *versus* human drug levels when the disposition of the agent differs markedly between species, particularly when an agent’s activity is predicted to be dependent upon maintaining drug levels of above a threshold concentration for a specific duration of time. As an example, the PPTP/C evaluated the HDAC inhibitor entinostat (Kurmasheva et al., 2019), an agent that has a short half-life in mice but a very long half-life (> 50 h) in humans (Knipstein & Gore, 2011). The anticancer activity for HDAC inhibitors is dependent upon exposure above a threshold concentration for a minimum time period (Wilson et al., 2013). To maintain entinostat trough concentrations in mice similar to those observed in patients required using entinostat doses that produced much higher Cmax entinostat concentrations and much higher systemic exposures (Kurmasheva et al., 2019).

### 4.2. Single-mouse trial (SMT) as an alternative experimental design

While the PPTP/C studies routinely used 50 *in vivo* models to represent ‘childhood cancer’, it is clear that a few models representing a tumor type (*e.g.*, neuroblastoma, ALL *etc.*) cannot encompass the genetic diversity that has been revealed through molecular characterization of these diseases. The challenge for future drug testing is how to address incorporation of adequate genetic/epigenetic diversity into preclinical drug testing. Under the Research to Accelerate Cures and Equity for Children Act (RACE for Children Act), FDA may mandate clinical assessment if the target of a drug being developed is substantially relevant to the growth and progression of childhood cancer. This will necessitate developing preclinical models representing molecular entities for each cancer type. However, response to a targeted drug may be context-specific (*e.g.*, BRAF V600E melanoma *vs* BRAF V600E colon cancer) (Ducreux et al., 2019; Patel et al., 2020), necessitating multiple models representing the same genetic variant.

Using conventional cohort testing designs (8–10 mice/treatment group), as in the PPTP/C studies, limits the number of different models tested, and hence limits the genetic subtypes that can be incorporated into a study within resource constraints. One approach to such limitations is to use an SMT design (Murphy et al., 2016). The objective of SMT design experiments is to identify agents with robust tumor-regressing activity against select PDX models, and because a single mouse is being used for each model the number of models against which an agent can be tested can be much greater. In the SMT design the readout for activity of the tested agent is the objective response category (*e.g.*, PD, SD, PR, CR) of each mouse bearing a different PDX model.

The validity of the SMT design was evaluated using a retrospective analysis of 2106 tumor-drug studies undertaken by the PPTP, in which the response of a tumor in one mouse, selected at random from the group, was compared to the median group response. This analysis showed that the SMT design accurately predicted response categories in 78 % of studies. Allowing for a deviation of ±1 response classification (*e.g.*, PR *versus* SD), the concordance was 95 %, for both solid tumor and ALL models. Further, the SMT analysis was accurate in identifying the antitumor activity of 66 of 67 drugs in terms of the ORR determined for each drug over a range of tumor models. Prospective studies with up to 90 ALL models and up to 50 solid tumor models have shown that SMT gives essentially similar results to conventional testing (Hingorani et al., 2020; Kendsersky et al., 2021; Lock et al., 2020). The SMT approach appears valid for identifying biologically meaningful antitumor activity (*i.e.*, tumor regression with long EFS), but it is not able to reliably identify agents that only slow tumor growth. For the latter type of agents, conventional testing is needed for assessing statistical significance between treatment and control groups (*e.g.*, in time to event). By eliminating the control group from the experimental design, the SMT approach can potentially incorporate up to 20-fold the number of models for evaluation of an agent, encompassing many diseases, or encompassing the genetic diversity of a given disease. With the availability of diagnosis and relapse models within the same tumor panel, there is a possibility to investigate tumor activity relevant to the disease state. Using a SMT design, it is possible to identify ‘exceptional responders’ for which results can be validated using conventional testing and can be potentially linked to the molecular characteristics of the model(s). While the SMT approach has obvious advantages, it is important to acknowledge that a prerequisite for its use is models that have stable growth characteristics that are well understood by the experimentalist.

The antitumor activity of an antibody conjugate (trastuzumab deruxtecan; DS-8201 A) serves as one example of the application of the SMT design (Hingorani et al., 2020). The sensitivity of 35 tumor models to trastuzumab deruxtecan varied considerably with some tumors progressing on treatment or having short event-free survival (EFS) as shown by the Kaplan-Meier analysis. Of note, four of five rhabdoid tumor models (both extracranial and CNS) remained in CR at the end of the observation period (20 weeks), indicating potential sensitivity of this disease to trastuzumab deruxtecan.

### 4.3. Combination therapy

Combination therapy is widely accepted as an essential component to curative therapy for pediatric cancers. A key question in pediatric drug development is which agents warrant prioritization for clinical testing in combination with other agents. The PPTP/C primarily contributed to addressing this question by attempting to identify agents with robust single agent activity that could then be combined with other agents to create potentially effective combinations. This approach to developing combinations of agents is based on additivity and depends on each of the agents in the combination contributing to cancer cell kill independently of the other agents. Palmer and colleagues have presented convincing evidence that the success of recent FDA-approved combination regimens can be explained in large part by additivity (Hwangbo et al., 2023). An alternative principle for developing combinations is synergy (supra-additivity) in which the benefit of an added agent is not dependent on its single agent activity but rather on its ability to enhance the activity of the other agent(s) in the combination. While clinically successful examples of combinations based on well-documented synergy are rare, the use of preclinical testing to identify synergistic combinations remains an area of focus for many research teams. When agents with limited single agent activity are used in combination regimens, the principle of synergy is inherently invoked, since by additivity the effect of adding an agent with little single agent activity will be minimal.

The primary metric used by the PPTP/C to evaluate combinations was based on the concept of therapeutic enhancement, previously termed therapeutic synergy (Corbett, Griswold Jr., Wolpert, Venditti, & Schabel Jr., 1979; Houghton, Morton, Gorlick, Lock, et al., 2010; Rose & Wild, 2004). Therapeutic enhancement can be claimed when the delay in time to event for the combination is significantly greater (*p* < 0.01) than the delay in time to event induced by either of the component agents used alone at their maximum tolerable dose. An important characteristic of therapeutic enhancement is that it acknowledges that if the doses of a combination’s component agents must be reduced from their single agent dose to create a tolerable regimen, then the combination is preferable to using the agents singly only if the combination is more effective than each of the agents used alone at their maximum tolerable dose. Evaluating for therapeutic enhancement is mechanism agnostic, and it may be present whether the interaction of the agents used in the combination is synergistic, additive, or antagonistic.

The PPTP/C also developed a method for formally evaluating for supra-additive or sub-additive effects for combinations of agents (Houghton, Morton, Gorlick, Lock, et al., 2010). The model-based analysis uses a linear regression model for time-to-event, with testing to determine whether there is a significant treatment interaction for the two-drug combination that is significantly different from 0. A significantly negative interaction term indicates sub-additivity, while a significantly positive interaction term indicates supra-additivity.

Combination testing by the PPTP/C primarily involved testing experimental agents with standard of care chemotherapy agents. Examples of agents for which this type of combination testing was performed include the WEE1 kinase inhibitor adavosertib (AZD1775) what was tested with irinotecan (Kolb et al., 2020), the ATR inhibitor berzosertib (formerly M6620, VX-970) that was tested with cisplatin (Kurmasheva et al., 2018), the mTOR inhibitor rapamycin that was tested with several standard of care agents (Houghton, Morton, Gorlick, Lock, et al., 2010), the HDAC inhibitor entinostat that was tested with agents used to treat rhabdomyosarcoma (Kurmasheva et al., 2019), the menin inhibitor VTP50469 that was tested with agents used in 3-drug induction therapy for ALL (Lock et al., 2023), and the PARP inhibitor talazoparib that was tested with temozolomide (Smith et al., 2015).

Adavosertib showed consistent therapeutic enhancement with irinotecan with the effect being most pronounced for osteosarcoma models (Kolb et al., 2020). By contrast, berzosertib and entinostat produced therapeutic enhancement in only a minority of experiments (Kurmasheva et al., 2018; Kurmasheva et al., 2019). Rapamycin showed therapeutic enhancement most consistently with cyclophosphamide, but therapeutic enhancement was also observed in some models with vincristine (Houghton, Morton, Gorlick, Lock, et al., 2010). Supra-additive effects were noted for some models, including a rhabdomyosarcoma model tested with vincristine.

The PPTP/C combination results with rapamycin helped support clinical development of the mTOR inhibitor temsirolimus in combination with standard chemotherapy regimens for rhabdomyosarcoma. In a phase 2 clinical trial in pediatric patients with relapsed/refractory solid tumors, temsirolimus did not show single agent activity against rhabdomyosarcoma (Geoerger et al., 2012). However, results of a randomized phase 2 clinical trial for patients with rhabdomyosarcoma at first relapse favored temsirolimus plus chemotherapy as being more effective than bevacizumab plus chemotherapy (Mascarenhas et al., 2019a). While the randomized phase 2 trial suggested potential benefit for the addition of temsirolimus to chemotherapy, a subsequent phase 3 trial showed no benefit for the addition of temsirolimus to standard chemotherapy for patients with newly-diagnosed intermediate risk rhabdomyosarcoma (Gupta et al., 2024). In this instance, the additivity model of Palmer and colleagues using clinical evidence for temsirolimus as being inactive against rhabdomyosarcoma better predicted the outcome of the phase 3 temsirolimus clinical trial than the preclinical data suggesting a potential supra-additive effect in a small number of models.

Combination testing of the menin inhibitor VTP50469 with standard chemotherapy agents against infant *KMT2A*-rearranged ALL models illustrates the evaluation of a combination that includes two active components (Lock et al., 2023). Both VTP50469 and the 3-drug chemotherapy regimen showed activity for the ALL PDX models studied, but the combination of VTP50469 with 3-drug chemotherapy induced both deeper responses and significantly prolonged time to event. Therapeutic enhancement for the addition of VTP50469 to 3-drug chemotherapy was documented for both of the two PDX models evaluated. These results support clinical development of menin inhibitors in combination with standard leukemia treatment regimens.

The combination of the PARP inhibitor talazoparib plus temozolomide provides an important cautionary example of the potential challenges of translating synergistic preclinical findings to clinical success. In a PPTP/C study focusing on Ewing sarcoma models, neither talazoparib nor temozolomide slowed tumor growth as single agents for these models (Smith, Reynolds, et al., 2015). Despite this lack of activity, the combination of the two agents administered for 5 days was extraordinarily effective, with complete responses in most Ewing sarcoma models tested and with some models not showing regrowth for more than 100 days. This remarkable degree of response was observed despite using a temozolomide dose that was approximately 4-fold lower than the standard temozolomide dose used for *in vivo* testing. A phase 1 study of talazoparib plus temozolomide in children with relapsed/refractory solid tumors found that the dose of temozolomide that could be administered with talazoparib was nearly 7-fold below the standard clinical dose of temozolomide. The dose-limiting toxicities in the phase 1 study were reversible neutropenia and thrombocytopenia. A phase 2 expansion cohort for patients with Ewing sarcoma failed to show objective responses to the clinically tolerable talazoparib-temozolomide combination that required use of the low temozolomide dose.

The PPTP/C experience with talazoparib and temozolomide recapitulates the experience with O^6^-benzylguanine and nitrosoureas. The preclinical synergistic activity observed for O^6^-benzylguanine administered with a nitrosourea could not be replicated in the clinic because of excessive toxicity in patients receiving both O^6^-benzylguanine and bichloroethyl nitrosourea (BCNU), which required a 5-fold reduction in the BCNU dose for the combination to be safely administered (Blumenthal et al., 2015; Friedman et al., 1992). The lesson from these experiences is that profound preclinical anticancer synergistic activity may fail to translate to clinical efficacy because the synergistic effect is equally applicable to normal tissues. Targeting one of the agents of a synergistic combination to tumor cells is a potential approach to overcoming the problem of synergistic toxicity, and nanoparticle-based drug formulations and antibody-drug conjugates are two drug delivery methods being studied for this purpose (Del Pozo et al., 2022; Kinneer et al., 2023; Mironova et al., 2024). That said, absent a clear rationale for tumor-selective synergistic activity, the risk of synergistic preclinical activity leading to failure in clinical testing because of synergistic toxicity must be considered.

## 5. Accessibility of genomic and preclinical testing data

Several informatics platforms described below are available that provide access to the genomic data and efficacy study results generated for the preclinical models used in the PPTP/PPTC/PIVOT programs (Table 9). Other resources listed in Table 9 host genomic data from pediatric tumor sequencing initiatives other than the PPTP/PPTC/PIVOT, such as the Treehouse Childhood Cancer Initiative, NCI’s Therapeutically Applicable Research to Generate Effective Treatments (TARGET) program, and the Kids First Data Resource Center. Collectively, these information resources provide useful data for selecting models for efficacy studies based on the expression of drug targets across different pediatric cancer types. Based on the experiences of the PPTP/C studies, transcriptional levels alone are often not sufficiently informative, so combining the genomic data with IHC labeling on tissue microarrays or slides has proven an effective strategy for model selection for efficacy studies.

### 5.1. PedcBioPortal

The PedcBioPortal was implemented using the cBioPortal platform (Cerami et al., 2012) and provides access to ‘omics’ data for over 260 preclinical pediatric models generated by the PPTP/C. Data from other pediatric cancer genomics initiatives, including NCI’s TARGET program are also accessible from this portal. Genomic data generated by the Hudson Alpha Institute for more than 250 additional PDX models from the PPTP/C and PIVOT programs have recently been made available through NCI’s Childhood Cancer Data Initiative.

### 5.2. St. Jude PeCan cloud resource

The St. Jude Pediatric Cancer (PeCan) Cloud Resource includes a model systems portal for access to data generated for *in vitro* and *in vivo* PDX models. Model data are organized as the Childhood Solid Tumor Network (CSTN; https://cstn.stjude.cloud/), Pediatric Brain Tumor Portal (PBTP; https://pbtp.stjude.cloud/), and Public Resource of Patient-derived and Expanded Leukemias (PROPEL; https://propel.stjude.cloud/).

### 5.3. PIVOT Portal

The PIVOT portal provides graphical and tabular summaries of the results of the efficacy studies performed by the PPTP/PPTC/PIVOT programs following publication and/or final review by industry partners. Data are organized by the agent tested and the pediatric cancer types. The tabular summaries are customizable and allow users to download study data.

### 5.4. Patient Derived Cancer Models Database (PDCM)

The PDCM resource is a searchable catalog of the PDX models, including the models generated by the PPTP/PPTC/PIVOT program investigators (Perova et al., 2023). Information available in this cancer model catalog includes the demographic and clinical characteristics of the patient whose tumor tissues were used to generate a model and a summary of the available data for the model.

### 5.5. Pediatric Patient Derived Xenograft (PDX) Explorer Database

This resource is a searchable catalog of the PDX models, including the models generated by the CPRIT (Cancer Prevention and Research Institute of Texas) program investigators (Rogojina et al., 2023). The clinical, genomics, protein data, and pathology images are integrated into the database, where investigators can explore functionalities, such as patient query, online analysis, and digital pathology visualization.

### 5.6. Childhood Cancer Data Initiative (CCDI)

The CCDI brings together demographic and genomic data from different pediatric and adolescent cancer studies into a platform for coordinated data sharing and analytics (Flores-Toro et al., 2023). Included in CCDI are genomic data for PDX models for pediatric patients generated by PPTP/C and PIVOT investigators. The NCI Genomic Data Commons (Zhang et al., 2021) is a related resource that provides researchers access to genomic data from pediatric patients.

## 6. Conclusions

The experience of the PPTP/C documents that it is feasible to establish large numbers of genomically characterized pediatric preclinical models and then to use the models assembled into disease-specific panels to test a wide range of novel anticancer agents. Given the diverse nature of childhood cancers and the biological heterogeneity that exists even within discrete diagnoses, the ability to test large numbers of models is an essential attribute of a contributory testing program. The PPTP/C successor, PIVOT (Pediatric *In Vivo* Testing Consortium), as well as the European pediatric preclinical testing program (ITCC-P4) are essential programs for meeting this challenge (Vassal et al., 2021).

An important innovation of the PPTP/C for pediatric preclinical testing was the introduction of the single mouse trial (SMT) design that markedly expands the breadth of tumor biology that can be evaluated through *in vivo* testing. The caveat that applies to the SMT design is that its objective is to identify agents able to induce robust tumor regressions, and that it cannot be applied to agents with more subtle therapeutic effects. That said, the agents that have had the largest impact in the treatment of childhood cancers are those capable of inducing substantial tumor regressions for solid tumors and complete remissions for leukemias. Hence, the SMT design is well suited to identify those agents most likely to have a meaningful clinical impact.

A deficiency of current pediatric preclinical testing programs is the paucity of pediatric preclinical models for testing agents that require an intact immune system. All of the PPTP/C *in vivo* testing was performed using PDX models grown in immunodeficient mice. While this testing approach can be used to evaluate some immuno-oncology agents, including CAR T-cells and antibody-drug congugates and bispecific T-cell engaging agents, the approach is not effective for testing agents like checkpoint inhibitors. While genetic mouse models for some childhood cancers (*e.g.*, MYCN-driven neuroblastoma) have been used to test agents that require an adaptive immune response, further research is required to develop robust panels of models across a diverse range of tumor types. Indeed, for many pediatric cancers such as Ewing sarcoma, no transgenic mouse model exists and spontaneous syngeneic tumors have not been developed. The application of pediatric preclinical testing for agents requiring adaptive immunity needs to be considered in the context of the limited response of childhood cancers to checkpoint inhibitors (outside of a few settings such as Hodgkin lymphoma and hypermutant pediatric tumors resulting from genetic conditions).

A crucial observation from the PPTP/C testing is that agents that are effective in the clinical setting show activity in pediatric preclinical models. The PPTP/C demonstrated that standard cytotoxic agents like vincristine, cyclophosphamide, and irinotecan showed activity in the solid tumor and leukemia models against which they are effective. Targeted agents like dasatinib for *BCR::ABL1* ALL, selumetinib for BRAF mutant gliomas, and PARP inhibitors for cancers with loss of homologous repair showed activity in relevant pediatric preclinical models. As a concern with reliance on *in vivo* preclinical testing for gating entry to clinical testing is the potential for false negative results in which a truly active clinical agent is ineffective in preclinical testing, these results are reassuring.

Agents with the same mechanism of action generally showed comparable activity patterns. As an example, multiple VEGF pathway inhibitors were tested, with the consistent finding across all models tested being the slowing of tumor growth without tumor regressions. Based on the PPTP/C experience, studying more than two or three agents in a single class for the same disease indication(s) is unlikely to add substantial knowledge about the utility of the class of agents for that indication. A potential suggested use case for preclinical testing is the comparison of agents in the same class to select the “optimal” agent for pediatric clinical testing. However, comparisons of agents within a target class in preclinical models are complicated by differences in mouse *versus* human drug exposures between agents that may obscure activity relationships. While such comparisons may be possible, exquisite attention to modeling drug exposures in the clinic to those in mice would be essential for developing meaningful assessments of the relative activity of the tested agents.

There were some agents tested by PPTP/C research teams that showed robust regressions (or remissions) in preclinical models for selected tumor types, but when tested in the clinical setting against these same tumor types showed no activity. These “false positive” results highlight the need for careful attention to dosing in preclinical experiments so that the drug levels in mice approximate those that are tolerated in patients. The experience with the AKR1C3-activated prodrug PR-104 illustrates this point well. In initial testing by the PPTP/C at a high dose of the agent, regressions and remissions were observed across a wide range of models, but when the dose was reduced to achieve more clinically relevant exposures, the only models responding were T-ALL models with elevated AKR1C3 expression. There were several examples of false positive results for ADCs tested by the PPTP/C, and more frequent dosing in preclinical testing compared to the clinic may have contributed to these results. As well, drug exposures were not evaluated for the ADCs tested by the PPTP/C, and future testing may benefit from confirming that ADC blood levels in mice bearing pediatric PDX models are in the range of those observed in patients.

The PPTP/C testing results show that many anticancer agents being developed for adult cancers lack tumor-regressing activity as single agents in pediatric preclinical models. This does not reflect that these agents are not effective anticancer agents when used in the correct setting, but rather, it illustrates the distinctive biology for pediatric cancers in comparison to carcinomas that predominate in adults and in comparison to the adult-specific hematologic malignancies (*e.g.*, multiple myeloma and CLL). Testing of proteasome inhibitors for pediatric solid tumors illustrates the point, as there was minimal activity observed against any of these models although the proteasome inhibitors are highly effective in adults with multiple myeloma, but similiarly not adult solid malignancies. Other classes of agents showing little or no tumor regressing activity, included the HDAC inhibitors, HSP90 inhibitors, and cell-signaling kinase inhibitors. These classes of agents lacking tumor-regressing activity in preclinical models have not shown meaningful clinical activity when studied in children. This observation supports the position that that preclinical testing can be used to screen agents for clinical testing and that agents lacking tumor-regressing activity can be deprioritized (in the absence of additional data supporting their evaluation).

The topic of deprioritizing agents unlikely to be effective has become especially salient due to the RACE for Children Act incorporated into the 2017 FDA Reauthorization Act (FDARA). The RACE Act requires the evaluation of new molecularly targeted drugs and biologics “intended for the treatment of adult cancers and directed at a molecular target substantially relevant to the growth or progression of a pediatric cancer”. Molecularly targeted pediatric cancer investigations are clinical trials that have the objective of evaluating “dosing, safety and preliminary efficacy to inform potential pediatric labeling”. Given the large universe of agents being developed for adult cancers, a process for deprioritization is essential for avoiding exposing children to agents with little likelihood of meaningful activity (but with the potential for producing adverse effects).

The experience of the PPTP/C demonstrates that a systematic approach to pediatric preclinical testing can provide data that are useful in identifying novel agents most likely to be effective in treating children with cancer as well as in identifying those highly unlikely to show activity in pediatric cancer patients. As a multitude of new agents continue to enter clinical evaluation each year, pediatric preclinical testing through the NCI PIVOT program (the successor of the PPTP/C) and the ITTC-P4 platform and other avenues is more important than ever.

## Figures and Tables

**Table 1:** Summary of objective response measure results for agents evaluated by the PPTP/C.

	Total Models	Solid Tumor	Acute Lymphoblastic Leukemia

Models tested	3382	2486	887
ORR	21 %	15%	37 %
CR/MCR%	15 %	9 %	29 %
PD2 %	13 %	14 %	9 %
PD1 %	56 %	64 %	34 %
Total PD%	76 %	82 %	61 %

Objective Response Measure (ORM) results for 144 agents tested by the PPTP/Care summarized. Most agents were tested against 40–50 models in tumor panels for acute lymphoblastic leukemia (ALL), osteosarcoma, neuroblastoma, brain tumors (*e.g.*, glioblastoma, medulloblastoma, atypical teratoid rhabdoid tumors), rhabdomyosarcoma, Ewing sarcoma, and Wilms tumor. The overall objective response rate (ORR) includes the percentage of treated models with partial response (PR), complete response (CR), or maintained CR (MCR). The percentage of models with CR/MCR% documents a higher level of tumor regression/remission. Progressive Disease 2 (PD2), and Progressive Disease 1 (PD1) ORM categories are provided separately for solid tumor models and acute lymphoblastic leukemia (ALL) models. PD2 indicates progressive disease with substantial slowing of tumor growth (2-fold extension of time to event compared to control animals), while PD1 indicates progressive disease with limited slowing of tumor growth. Definitions for Objective Response Measure categories are provided in Section 3.0.

**Table 2:** Objective response rates for HSP90, HDAC, and proteasome inhibitors.

	HSP90 Inhibitors		HDAC Inhibitors		Proteasome Inhibitors	
			
	Solid Tumor	Acute Lymphoblastic Leukemia	Solid Tumor	Acute Lymphoblastic Leukemia	Solid Tumor	Acute Lymphoblastic Leukemia

Models tested	75	20	73	15	33	16
ORR	3 %	5 %	1 %	13 %	6 %	25 %
CR/MCR%	1 %	0 %	1 %	13 %	0 %	13 %
PD2 %	8 %	30 %	10 %	7 %	3 %	0 %
PD1 %	85 %	55 %	86 %	73 %	91 %	75 %
Total PD%	97 %	85 %	99 %	80 %	94 %	75 %

Objective Response Measure (ORM) results are provided for three HSP90 inhibitors [alvespimycin (17-DMAG), onalespib (AT13387), and ganetespib (STA9090)], for three HDAC inhibitors [vorinostat, quisinostat (JNJ26481585), and entinostat] and for two proteasome inhibitors (bortezomib and ixazomib). See Table 1 for a description of the ORM categories. References to primary publications for each agent are provided in the text.

**Table 3:** Objective response measure (ORM) percentages forVEGFR-targeted and non-VEGFR-targeted agents.

	Solid Tumor		Acute Lymphoblastic Leukemia	
		
	VEGFR2-Targeted	Non-VEGFR2-Targeted	VEGFR2-Targeted	Non-VEGFR2-Targeted

Models tested	158	2328	24	863
ORR	4 %	16 %	8 %	38 %
CR/MCR%	1%	10 %	0 %	30 %
PD2 %	41 %	12 %	13 %	8 %
PD1 %	47 %	65 %	79 %	33 %
Total PD%	94 %	81 %	92 %	60 %

Objective Response Measure (ORM) results are provided for solid tumor models and for leukemia models for six VEGFR2-targeted kinase inhibitors studied by the PPTP/C: cediranib, sunitinib, sorafenib, pazopanib, regorafenib, and cabozantinib. The ORM results are also provided for all non-VEGFR2-targeted agents studied by the PPTP/C. The key findings are the low ORR and the high PD2 % rate for VEGFR2-targeted agents compared to non-VEGFR2-targeted agents. See Table 1 for a description of the ORM categories. References to primary publications for each agent are provided in the text.

**Table 4:** Molecularly targeted agents studied by the PPTP/C with single agent activity.

Agent	Agent Target	# Tested	Objective Response Rate	Responsive Models

Dasatinib	BCR::ABL1	43	2%	*BCR*::*ABL1* ALL
Sunitinib	VEGFR2 & FLT3	45	9 %	*FLT3*-mutated ALL
Selumetinib	MEK	46	4 %	*BRAF* V600E mutated glioma
VTP-50469	Menin-KMT2A	16	38 %	*KMT2A*-rearranged infant ALL
Talazoparib	PARP	44	7 %	*PALB2*-mutant Wilms tumor

Objective response rates are provided for molecularly targeted agents for which single agent activity was observed in testing against PPTP/C preclinical models. Testing against multiple PPTP/C tumor panels was performed for dasatinib, sunitinib, selumetinib, and talazoparib, with a low overall objective response rate outside of models with genomic alterations relevant to the agents’ targets, as indicated in the table. The menin inhibitor VTP-50469 was tested against selected ALL models and Ewing sarcoma models, with activity observed only for KMT2A-rearranged infant ALL models. References to primary publications for each agent are provided in the text.

**Table 5:** Objective response rates observed for antibody-drug conjugates against PPTP/C preclinical models.

Agent	Target	Tumor Types	Solid Tumor		Acute Lymphoblastic Leukemia	
		
			# Tested	ORR	# Tested	ORR

Coltuximab Ravtansine (SAR3419)	CD19	ALL	0	NA	3	100 %
Denintuzumab mafodotin (SGN-CD19A)	CD19	ALL	0	NA	7	71 %
Pivekimab sunirine (IMGN632)	CD123	ALL	0	NA	8	75 %
Zilovertamab vedotin (VLS-101)	ROR1	ALL, EWS	5	40 %	7	29 %
VLS-211	ROR1	ALL, EWS	5	60 %	7	43 %
Lorvotuzumab mertansine (IMGN901)	CD56	OS, RMS, NB, Rhabdoid, CNS, Wilms	25	36 %	0	NA
Glembatumumab vedotin (CDX-011)	GPNMB	OS, RMS	8	38 %	0	NA
Telisotuzumab vedotin (ABBV-399)	MET	RMS	4	0 %	0	NA
Samrotamab vedotin (ABBV-085)	LRRC15	OS	7	29 %	0	NA
Rovalpituzumab tesirine (Rova-T)	DLL3	NB	10	20 %	0	NA
ADCT-701	DLK1	NB	12	58 %	0	NA
Trastuzumab deruxtecan (DS-8201A)	HER2	OS, EWS, RMS, NB, Rhabdoid, Wilms	39	56 %	0	NA
m276-PBD	CD276 (B7-H3)	OS, EWS, RMS, NB, Rhabdoid, Wilms	59	93 %	0	NA
Vobramitamab duocarmazine (MGC018)	CD276 (B7-H3)	NB	10	30 %	0	NA
All Models Tested			**184**	**59 %**	**32**	**59 %**

The number of solid tumor and leukemia models studied as well as the objective response rates (ORR) are shown for ADCs studied by the PPTP/C for solid tumor models and for acute lymphoblastic leukemia (ALL) models. Abbreviations: OS, osteosarcoma; EWS, Ewing sarcoma; RMS rhabdomyosarcoma; CNS, central nervous system tumors; NB, neuroblastoma; NA, not applicable. References to primary publications for each agent are provided in the text.

**Table 6:** Activity observed for mitotic kinase inhibitors against PPTP/C preclinical models.

Agent	Target	Solid Tumor		Acute Lymphoblastic Leukemia	
		
		# Tested	ORR	# Tested	ORR%

Ispinesib	KSP5	30	23 %	8	75 %
Alisertib	Aurora A kinase	41	27 %	6	100 %
GSK923295A	CENP-E	37	32 %	6	83 %
Volasertib	PLK1	34	9 %	8	63 %
ALL Models Tested		**142**	**23 %**	**28**	**79 %**

The number of solid tumor and acute lymphoblastic leukemia models studied as well as their objective response rates (ORR) are shown for each of the mitotic kinase inhibitors studied by the PPTP/C. Results are shown separately for solid tumor and for acute lymphoblastic leukemia models. References to primary publications for each agent are provided in the text.

**Table 7:** Activity observed for PI3K pathway-targeted agents against PPTP/C models.

Agent	TARGET	Solid Tumor			Acute Lymphoblastic Leukemia		
	
		# Tested	PD2%	ORR	# Tested	PD2%	ORR

GSK690693	AKT	41	5 %	2%	8	0 %	0 %
MK-2206	AKT	30	3 %	0 %	8	50 %	0 %
Pilaralisib (XL147)	Pan-PI3K	38	13 %	0 %	7	29 %	0 %
Copanlisib	Pan-PI3K	6	0 %	0 %	0		
Acalisib (GS-9820)	PI3Kδ	0	NA	NA	7	0 %	0 %
Duvelisib	PI3Kδ	0	NA	NA	30	0 %	3 %
Voxtalisib (XL765)	PI3K/mTOR	2	0 %	0 %	0		
Sapanisertib (INK128)	MTOR kinase	31	19 %	0 %	7	0 %	0 %
AZD8055	MTOR kinase	38	13 %	0 %	7	0 %	0 %
Rapamycin	MTOR	37	30 %	14 %	8	25 %	38 %

Objective response rates (ORR) and rates of PD2 responses (progressive disease with time to event delayed 2-fold compared to control) are shown for agents targeting the PI3K pathway. Most agents were tested broadly against multiple PPTP/C tumor panels, while voxtalisib was only tested against GBM models, copanlisib was tested against only osteosarcoma models, and duvelisib and acalisib were tested against only leukemia models. Abbreviations: NA, not applicable. References to primary publications for each agent are provided in the text.

**Table 8:** Objective response measure (ORM) results for IGF-1R targeting monoclonal antibodies.

	Solid Tumor	Ewing	Osteosarcoma	Rhabdomyosarcoma	Neuroblastoma

#					
Models	70	10	11	10	11
ORR	6 %	10 %	18 %	10 %	0 %
PD2%	29 %	20 %	36 %	60 %	27 %

Objective response rates (ORR) and rates of PD2 responses (progressive disease with time to event delay 2-fold compared to control) are shown for the two IGF-1R targeting monoclonal antibodies studied by the PPTP/C: robatumumab (19D12) and cixutumumab (IMC-A12). Results are shown for all solid tumor preclinical models tested as well as for models for selected solid tumor types. References to primary publications for each agent are provided in the text.

**Table 9:** Data sources for pediatric cancers and preclinical models.

Resource name	Data types	PPTP/C model data included?	URL	Reference

PedcBioPortal	Mutations, gene expression, structural variants, copy number alterations	Yes	https://pedcbioportal.org	(Rokita et al., 2019)
NCI’s Genomic Data Commons	Clinical and genomic data	No	https://portal.gdc.cancer.gov/	(Zhang et al., 2021)
NCI’s Childhood Cancer Data Initiative	Demographic, clinical, and genomic data	Yes	https://ccdi.cancer.gov/explore	(Flores-Toro et al., 2023)
Kids First	Clinical, genetic, and genomic data	No	https://kidsfirstdrc.org/	
Treehouse Childhood Cancer Initiative	Genomic data for PDX models, patient tumors, and cell lines	No	https://treehousegenomics.ucsc.edu/	
St. Jude PeCan Cloud Models Portal	Mutations, variants, gene expression, histology	Yes	https://models.stjude.cloud/	(McLeod et al., 2021)
PIVOT Portal	Efficacy study results	Yes	http://preclinicalpivot.org	
Patient Derived Cancer Models Database	PDX model details	Yes	https://www.cancermodels.org/	(Perova et al., 2023)
Pediatric PDX Explorer	Mutations, gene expression, methylation analysis, pathology images	Yes	https://datacommons.swmed.edu/cce/ppdxe/data.php	(Rogojina et al., 2023)

## Data Availability

Data are available from Zenodo (https://zenodo.org/records/13871579).

## Abbreviations:

NCI: National Cancer Institute
PPTP: Pediatric Preclinical Testing Program
PPTC: Pediatric Preclinical Testing Consortium
PIVOT: Pediatric Preclinical *In Vivo* Testing
FDA: Federal Drug Administration
FDARA: FDA reauthorization act
COG: Children’s Oncology Group
SWOG: Southwest Oncology Group
RACE: Research to Accelerate Cures and Equity for Children Act
ITTC-P4: Innovative Therapies for Children with Cancer Pediatric Preclinical Proof-of-Concept Platform
PDX: patient-derived xenograft
IHC: immunohistochemistry
SMT: single mouse trial
MTD: maximum tolerated dose
HRD: homologous recombination deficiency
CAR: chimeric antigen receptor
ADC: antibody drug conjugate
MAB: monoclonal antibody
NGS: next generation sequencing
MMAE: Monomethyl auristatin E
MMAF: Monomethyl auristatin F
CRISPR: clustered regularly interspaced short palindromic repeats
NOD/SCID: *NOD*/ShiLtSz-Prkdc^*scid*^
BCNU: bichloroethyl nitrosourea
^123^I-MIBG: ^123^I-metaiodobenzylguanidine
ORR: objective response rate
MCR: maintained complete response
CR: complete response
PR: partial response
SD: stable disease
PD: progressive disease
PD1: progressive disease without tumor growth delay
PD2: progressive disease with tumor growth delay
TCGA: The Cancer Genome Atlas
PDCM: Patient-Derived Cancer Models
CCDI: Childhood Cancer Data Initiative
CSTN: Childhood Solid Tumor Network
CPRIT: Cancer Prevention and Research Institute of Texas
PDCM: Patient-Derived Cancer Models Database
PeCan: Pediatric Cancer
TARGET: Therapeutically Applicable Research to Generate Effective Treatments
ALL: acute lymphoblastic leukemia
T-ALL: T-cell acute lymphoblastic leukemia
CNS: central nervous system
Ph+: Philadelphia chromosome positive
GBM: glioblastoma
CLL: chronic lymphocytic leukemia
ccRCC: clear cell renal cell carcinoma
VEGF: vascular endothelial growth factor
HSP90: heat shock protein 90
HDAC: Histone deacetylase
VEGFR2: Vascular endothelial growth factor receptor 2
KIT: KIT proto-oncogene receptor tyrosine kinase
AML: acute myeloid leukemia
FLT3: fms related receptor tyrosine kinase 3
VHL: von Hippel-Lindau tumor suppressor
HIFA: hypoxia inducible factor 1 subunit alpha
ALK: ALK receptor tyrosine kinase
BRAF: B-Raf proto-oncogene serine/threonine kinase
NOTCH1: notch receptor 1
BCR: BCR activator of RhoGEF and GTPase
ABL1: ABL proto-oncogene 1 non-receptor tyrosine kinase
KMT2A: lysine methyltransferase 2A
SRC: SRC proto-oncogene non-receptor tyrosine kinase
RTK: Receptor tyrosine kinase
FLT3: MEK1/2 mitogen-activated protein kinase kinase 1 and 2
JAK1 and 2: Janus kinase 1 and 2
SYK: spleen associated tyrosine kinase
PARP: poly(ADP-ribose) polymerase
PALB2: partner and localizer of BRCA2
BRCA1 and 2: BRCA1 and 2 DNA repair associated
BARD1: BRCA1 associated RING domain 1
PI3K: phosphatidylinositol-4,5-bisphosphate 3-kinase
PIM1/2/3: Pim-1/2/3 proto-oncogene serine/threonine kinase
CD30 (TNFRSF8): TNF receptor superfamily member 8
CD22: CD22 molecule
CD19: CD19 molecule
CD56 (NCAM1): neural cell adhesion molecule 1
CD123 (IL3RA): interleukin 3 receptor subunit alpha
B7-H3 (CD276): CD276 molecule
DLL3: delta like canonical Notch ligand 3
DLK1: delta like non-canonical Notch ligand 1
GPNMB: glycoprotein nmb
HER2 (ERBB2): erb-b2 receptor tyrosine kinase 2
LRRC15: leucine rich repeat containing 15
ROR1: receptor tyrosine kinase like orphan receptor 1
ERBB3: erb-b2 receptor tyrosine kinase 3
GPC2: glypican 2
GPC3: glypican 3
GD2 (B4GALNT1): beta-1,4-*N*-acetyl-galactosaminyltransferase 1
IL1RAP: interleukin 1 receptor accessory protein
ENPP1: ectonucleotide pyrophosphatase/phosphodiesterase 1
STEAP1: STEAP family member 1
CENPE: centromere protein E
PLK1: polo like kinase 1
KSP (KIF11): kinesin family member 11
BCL2: BCL2 apoptosis regulator
MDM2: MDM2 proto-oncogene
BCL-xL (BCL2L1): BCL2 like 1
BCL-W (BCL2L2): BCL2 like 2
MCL1: MCL1 apoptosis regulator
BCL2: family member
TORC1 and 2 (CRTC1 and 2): CREB regulated transcription coactivator 1 and 2
mTOR: mechanistic target of rapamycin kinase
PIK3CA: phosphatidylinositol-4,5-bisphosphate 3-kinase catalytic subunit alpha
ATP: adenosine triphosphate
PTEN: phosphatase and tensin homolog
IGF1: insulin like growth factor 1
IGF2: insulin like growth factor 2
IGF1R: insulin like growth factor 1 receptor
INSR: insulin receptor
KEAP: kelch like ECH associated protein 1
NRF2: NFE2 like bZIP transcription factor 2
TSC1: TSC complex subunit 1
TSC2: TSC complex subunit 2
AKR1C3: aldo-keto reductase family 1 member C3
WEE1: WEE1 G2 checkpoint kinase
MYCN: MYCN proto-oncogene
bHLH: transcription factor
